# BioTransformer: a comprehensive computational tool for small molecule metabolism prediction and metabolite identification

**DOI:** 10.1186/s13321-018-0324-5

**Published:** 2019-01-05

**Authors:** Yannick Djoumbou-Feunang, Jarlei Fiamoncini, Alberto Gil-de-la-Fuente, Russell Greiner, Claudine Manach, David S. Wishart

**Affiliations:** 1 Department of Biological Sciences, University of Alberta, Edmonton, AB T6G 2E9 Canada; 2 INRA, Human Nutrition Unit, Université Clermont Auvergne, 63000 Clermont-Ferrand, France; 3 Department of Food and Experimental Nutrition, School of Pharmaceutical Sciences, University of São Paulo, São Paulo, Brazil; 4 Department of Information Technology, CEU San Pablo University, Madrid, Spain; 5 Department of Computing Science, University of Alberta, Edmonton, AB T6G 2E8 Canada; 6 Alberta Machine Intelligence Institute, University of Alberta, Edmonton, AB T6G 2E8 Canada

**Keywords:** Metabolism prediction, Metabolite identification, Biotransformation, Microbial degradation, Mass spectrometry, Machine learning, Knowledge-based system, Structure-based classification, Metabolic pathway, Enzyme-substrate specificity

## Abstract

**Background:**

A number of computational tools for metabolism prediction have been developed over the last 20 years to predict the structures of small molecules undergoing biological transformation or environmental degradation. These tools were largely developed to facilitate absorption, distribution, metabolism, excretion, and toxicity (ADMET) studies, although there is now a growing interest in using such tools to facilitate metabolomics and exposomics studies. However, their use and widespread adoption is still hampered by several factors, including their limited scope, breath of coverage, availability, and performance.

**Results:**

To address these limitations, we have developed BioTransformer, a freely available software package for accurate, rapid, and comprehensive in silico metabolism prediction and compound identification. BioTransformer combines a machine learning approach with a knowledge-based approach to predict small molecule metabolism in human tissues (e.g. liver tissue), the human gut as well as the environment (soil and water microbiota), via its metabolism prediction tool. A comprehensive evaluation of BioTransformer showed that it was able to outperform two state-of-the-art commercially available tools (Meteor Nexus and ADMET Predictor), with precision and recall values up to 7 times better than those obtained for Meteor Nexus or ADMET Predictor on the same sets of pharmaceuticals, pesticides, phytochemicals or endobiotics under similar or identical constraints. Furthermore BioTransformer was able to reproduce 100% of the transformations and metabolites predicted by the EAWAG pathway prediction system. Using mass spectrometry data obtained from a rat experimental study with epicatechin supplementation, BioTransformer was also able to correctly identify 39 previously reported epicatechin metabolites via its metabolism identification tool, and suggest 28 potential metabolites, 17 of which matched nine monoisotopic masses for which no evidence of a previous report could be found.

**Conclusion:**

BioTransformer can be used as an open access command-line tool, or a software library. It is freely available at https://bitbucket.org/djoumbou/biotransformerjar/. Moreover, it is also freely available as an open access RESTful application at www.biotransformer.ca, which allows users to manually or programmatically submit queries, and retrieve metabolism predictions or compound identification data.

**Electronic supplementary material:**

The online version of this article (10.1186/s13321-018-0324-5) contains supplementary material, which is available to authorized users.

## Introduction

Metabolism is key to the production of energy (catabolism), the generation of cellular building blocks (anabolism) as well as the activation, detoxification, and elimination of metabolic by-products or xenobiotics. Over the past 100 years, considerable effort has gone into determining the precise molecular details of primary metabolism—i.e. the metabolic processes associated with the production and breakdown of essential metabolites (e.g. lipids, amino acids, and steroids) [1]. Unfortunately, somewhat less effort has been devoted to the characterization or understanding of non-essential or secondary metabolism and non-essential metabolites, partly due to their much higher number, and greater structural complexity, compared to primary metabolites.

**Fig. 1:**
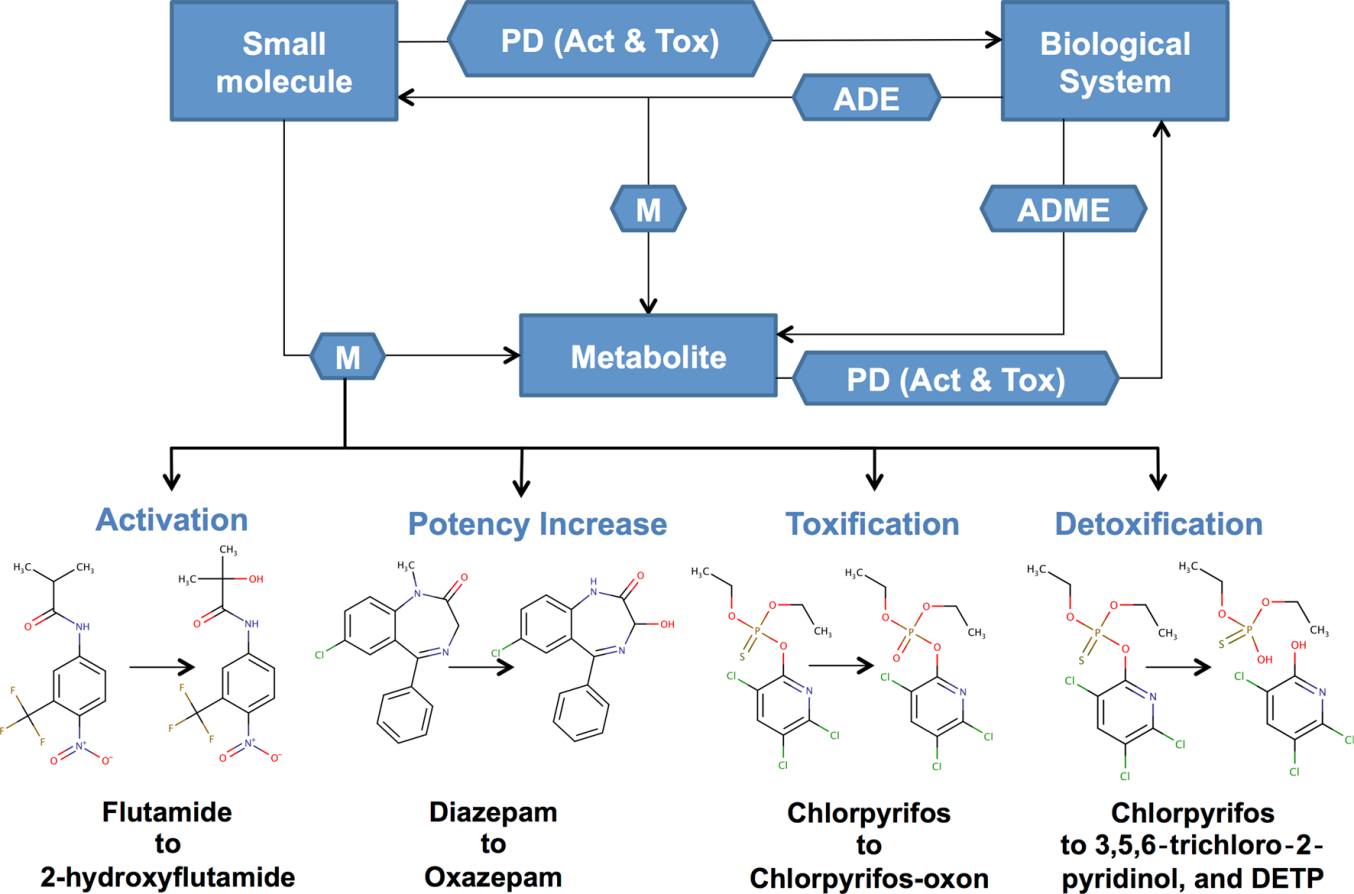
Effects of metabolism on the pharmacokinetics and pharmacodynamics of small molecules. This figure illustrates how metabolism of a xenobiotic can alter its pharmacodynamics (PD), including pharmacological activity (Act), and toxicological effects (Tox). Moreover, the nature of the resulting metabolites can influence their involvement in pharmacokinetic processes (i.e. *ADME* absorption, distribution, metabolism, excretion). *DETP* diethylthiophosphate

Non-essential metabolites include metabolites generated through the activation, detoxification and elimination of metabolic by-products or xenobiotics. Xenobiotics are compounds such as pharmaceuticals and personal care products (PPCPs), pesticides, plant or food compounds, food additives, surfactants, solvents, and other man-made or biologically foreign substances. They constitute the largest portion of the human chemical exposome of which more than 95% remain unknown or largely uncharacterized [2, 3]. In many cases, non-essential metabolites are the products of promiscuous or non-specific enzymatic reactions [4, 5], microbial or gut metabolism [6, 7], liver-based phase I metabolism (oxidation, reduction or hydrolysis) or general phase II metabolism (conjugation). Metabolism is known to significantly influence the pharmacokinetics and pharmacodynamics of xenobiotics and their derivatives within a biological system [8] (Fig. 1). Moreover, given the diversity of biological systems that constitute our environment, it is clear that understanding xenobiotic metabolism is critical to accurately linking chemistry and biology, and understanding the interactions between those biological systems and the environment.

**Fig. 2:**
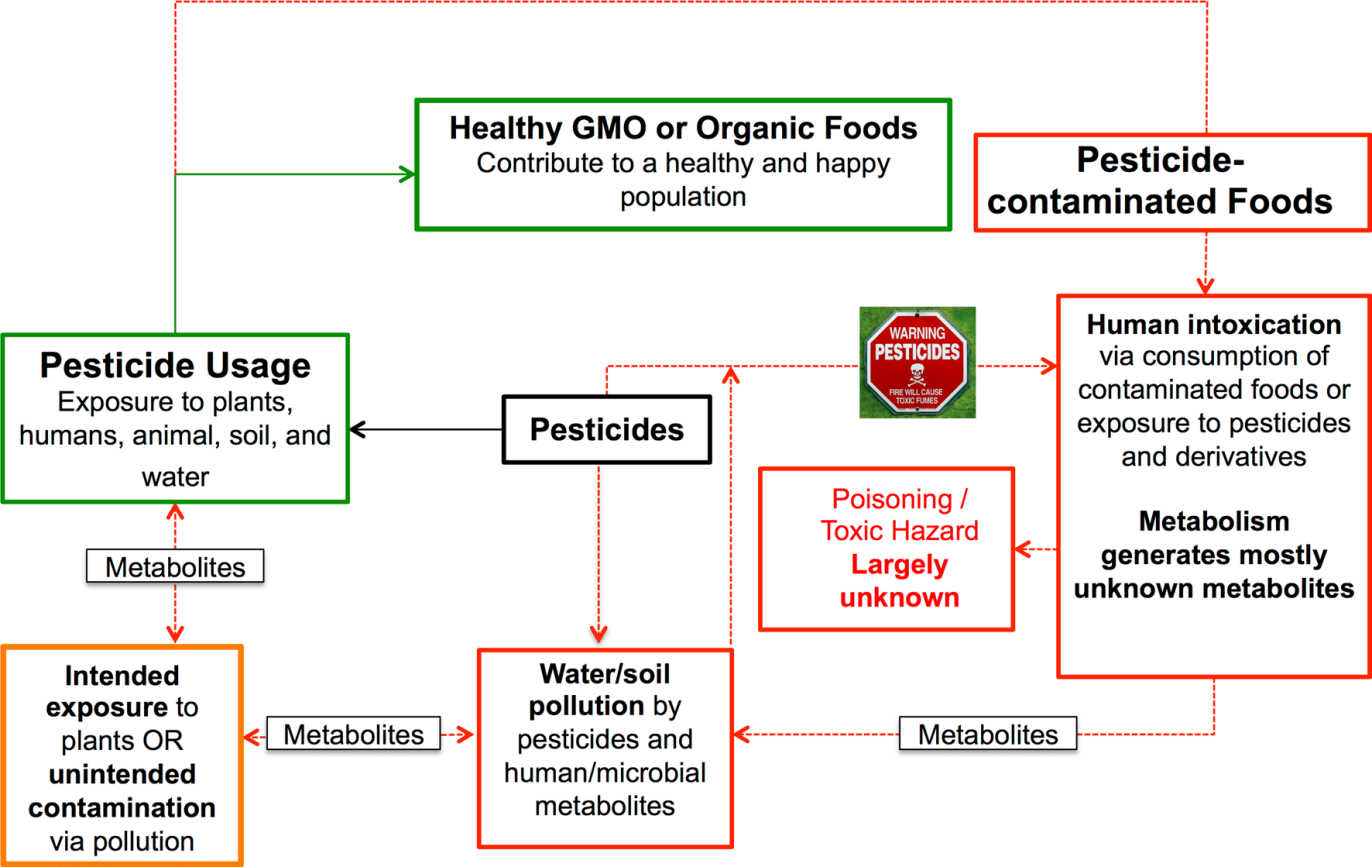
The life cycle of a xenobiotic: this figure partly illustrates the circulation, transformations, and effects of pesticides in humans and the environment. These substances can enhance crop protection, thereby increasing the yield of healthy foods. However, they can also contaminate soil and water meaning that they can find their way into non-target organisms, including humans. Moreover, upon exposure to pesticides humans usually generate and excrete pesticide metabolites into the environment, which can also contaminate soil and aquatic environments. Some of these metabolites, and their microbial degradation products have been isolated from water and food samples, showing that they can re-enter the human food chain [15, 16, 21]. This cycle is applicable to other types of xenobiotics, including pharmaceuticals, and personal care products, among others

Figure 2 partially describes the “life cycle of a xenobiotic”, using pesticides as an example. Pesticides can be used to protect plants against insect pests, waterborne ailments, other plant competitors and parasites, thus enabling the production of larger amounts of high quality food products, while using less land [9]. In this regard, pesticides contribute to a healthier way of life. However, exposure to pesticides through inhalation (e.g. by farmworkers), skin contact, or ingestion of contaminated harvested products is known to cause harmful effects (Fig. 2). For instance, the organophosphate pesticide Chlorpyrifos (see Additional file 1) can be activated in humans to become the carcinogenic substance Chlorpyrifos-oxon, through CYP450-catalyzed desulfurization [10]. Moreover, exposure to Chlorpyrifos has been linked to a decrease in the population of probiotic *Lactobacillus* and *Bifidobacterium* species in the gut microbiota of rats [11]. Interestingly, human CYP450-catalyzed metabolism of Chlorpyrifos can also lead to the generation of the inactive metabolites 3,5,6-trichloro-2-pyridinol, and diethyl phosphorothioate (see Additional file 1), via *O*-dearylation [10].

Once released from the human body into the environment, the pool of xenobiotics and their derivatives often contaminate soil and water, where they are often further degraded by soil and/or aquatic microbes. The resulting metabolites, which are mostly unknown, can affect soil/water microbial diversity, and soil fertility [12] and even re-enter the food chain [13, 14] (Fig. 2). Such a metabolic “life cycle” is applicable to other chemicals, such as pharmaceuticals, food additives, and other man-made products, as highlighted by a steadily increasing number of independent studies [15, 16]. For these reasons, the characterization of xenobiotic metabolites, which has long been vitally important to the pharmaceutical industry [5], has become increasingly more important to the pesticide industry [17] and to the fields of metabolomics [18], exposomics [3], and environmental sciences [19, 20].

The characterization or identification of xenobiotic metabolites from biological or environmental samples is quite difficult and is not unlike natural product identification or dereplication [22]. It can take months or even years to purify and positively identify a metabolite using standard analytical techniques. As a result, there has been a growing focus on using in silico strategies to help with this process. Indeed, over the past two decades, a number of very effective computational tools have been developed to predict the metabolism of xenobiotics—especially drugs. These computer programs typically require a starting parent molecule and employ pattern recognition techniques along with hand-made rules or machine learning algorithms to identify: (1) a site of reaction or a site of metabolism (SoM) within the molecule; and/or (2) a set of chemical products resulting from a biotransformation at the specific SoM. Most in silico metabolism prediction tools are quite specific to certain classes of reactions or metabolic processes, such as phase I (only) or phase II (only) reactions. Some in silico metabolism predictors, such as SMARTCyp [23, 24] and isoCYP [25], are limited to predicting phase I metabolism (or a portion of phase I metabolism), while others are more comprehensive (e.g. Meteor Nexus—Lhasa Limited, UK) [26] and SyGMa [27] cover a broad range of phase I and phase II biotransformations. Some programs are commercial such as Meteor Nexus, MetabolExpert (CompuDrug, Bal Harbor, FL, USA) [28] and ADMET Predictor (Simulation Plus, Lancaster, CA, USA) [29], while others are freely available either as web services (e.g. XenoSite [30] or as freely accessible standalone software packages (e.g. SMARTCyp). Most of these tools are focused on mammalian metabolism (e.g. Meteor Nexus). In comparison, a smaller number are targeted towards environmental microbial degradation. Such tools include enviPath, a complete redesign of the EAWAG-BBD/PPS, which in turn originates from the UM-BBD and UM-PPS systems [31–34]. The necessity for such tools, along with the aforementioned developments, have motivated certain mass spectrometry vendors to integrate metabolism prediction tools into their data processing systems [35, 36]. Such integration often simplifies the discovery of unknown metabolites, even at low concentration levels.

Unfortunately, even with the growing abundance of in silico metabolism prediction tools, there continues to be a number of significant limitations, especially with regard to their performance, their scope and their accessibility. In particular: (1) very few tools predict more than the SoMs; (2) none of the tools combine phase I, II, gut microbial metabolism, promiscuous enzymatic metabolism, and environmental microbial metabolism together; (3) many tools suffer from poor performance [37]; (4) almost all of the tools were developed and trained on drug molecules and were not adapted for non-drug xenobiotics; (5) only a small number of tools provide predicted structures in a downloadable or shareable format, and those that do place severe restrictions on their distribution; (6) almost none of the existing tools are open access or open source; and (7) very few of the tools make their databases or training sets available. These limitations have slowed the development of in silico metabolism prediction software and have also restricted the field to a tiny number of applications, mainly in the pharmaceutical industry.

Addressing these limitations and extending the capabilities of in silico metabolism prediction software could lead to substantial benefits in many other scientific disciplines including, but not limited to, analytical chemistry, natural product chemistry, agricultural and nutrition science, environmental chemistry, exposomics and metabolomics. Potential applications might include the in silico expansion of chemical databases of drugs (e.g. DrugBank [38]), food compounds (e.g. FooDB [39]), phytochemicals (e.g. PhytoHub [40]), environmental contaminants (e.g. ContaminantDB [41], T3DB [42], the CompTox Database [43]), organism-specific metabolites (e.g. HMDB [2], ECMDB [44], YMDB [45]), and other chemicals of biological interest (e.g. ChEBI [46], KEGG [47]). In fact, a notable effort carried by Jeffryes et al., has led to the development of the Metabolic In silico Network Expansion (MINEs) databases. The MINE databases contain close to 600,000 metabolites from compounds derived from KEGG [47], EcoCyc [48], and YMDB [45]. The metabolites were generated computationally using reaction rules based on the Enzyme Commission classification system [49], and the Biochemical Network Integrated Computational Explorer (BNICE) algorithm [50]. Jefrryes et al. reported that 93% of the computationally generated putative metabolites starting from KEGG compounds were not found in PubChem, the largest publicly accessible chemical database. Therefore, we anticipate that in silico expansions of the aforementioned databases using BioTransformer, will lead to the discovery of new exposure biomarkers, new bioactive metabolites, and consequently to the development of better drugs and consumer products (e.g. food, household and cosmetic products). This may ultimately lead to improved toxicology assessment, and the advancement of precision medicine [51] Moreover, the integration of predicted metabolites with their corresponding in silico predicted MS spectra could facilitate the identification of unknowns using metabolite identification tools such as CFM-ID [52–54], and MetFrag [55]. This would, in turn, help to further identify and characterize the so-called “dark matter” of the metabolome, which consists of the chemical signatures or molecules that remain uncharacterized or undiscovered [56].

Here, we present BioTransformer, an open access software tool, and freely accessible web service for accurate, and comprehensive in silico metabolism prediction and metabolite identification. It has been specifically designed to address essentially all of the shortcomings previously identified with existing in silico metabolism prediction tools. In particular, BioTransformer is freely available and furthermore its databases and predictions are free to download and use. It consists of two components: a metabolism prediction tool, and a metabolite identification tool. BioTransformer’s metabolism prediction tool (BMPT) generates predicted metabolite structures in standard electronic formats, and it provides comprehensive metabolite predictions. BMPT covers a wide range of molecular classes. In particular, BMPT combines a knowledge (or rule)-based approach with a machine learning approach to predict (1) human CYP450-calyzed phase I metabolism of xenobiotics, (2) human gut microbial metabolism, (3) phase II metabolism, (4) promiscuous enzymatic metabolism, and (5) environmental microbial metabolism of endogenous and exogenous compounds. For the prediction of CYP450 metabolism, BioTransformer makes use of CypReact [57], a tool for CYP450 substrate specificity prediction. BioTransformer also implements a set of rules provided by the EAWAG-BBD/PPS system [33] to predict the products of environmental microbial degradation. BioTransformer’s Metabolite Identification Tool (BMIT) builds upon the metabolite prediction tool, and can be used to identify metabolites of a given molecule that match a given set of masses or molecular formulas.

In addition to providing a description of BioTransformer, we also provide a detailed analysis of its performance, including a number of comparative analyses of BioTransformer against Meteor Nexus [26] and ADMET Predictor [29]. These analyses were done using the results of published studies on experimentally determined metabolites identified after specific exposures to drugs, foods, pesticides, and other xenobiotics by various mammalian species. We also describe the freely available BioTransformer RESTful web service, which allows users to freely predict and identify metabolites of diverse types of compounds, including but not limited to PPCPs, food compounds, phytochemicals, environmental contaminants/pollutants, as well as endogenous and other exogenous compounds. BioTransformer is available as an open access Java library at https://bitbucket.org/djoumbou/biotransformerjar. The JAR library can either be run as a command-line executable, or used as an imported library within a project. The BioTransformer web service is also freely accessible at www.biotransformer.ca.

## Methods

### Structure and implementation of BioTransformer

BioTransformer consists of a metabolism prediction tool (BMPT), and a metabolite identification tool (BMIT). The BMPT consists of five independent prediction modules called “transformers”, namely: (1) the Enzyme Commission based (EC-based) transformer, (2) the CYP450 (phase I) transformer, (3) the phase II transformer, (4) the human gut microbial transformer, and (5) the environmental microbial transformer. For the prediction of metabolites, BioTransformer implements two approaches, a rule-based or knowledge-based approach, and a machine learning approach. BioTransformer’s knowledge-based system consists of three major components: (1) a biotransformation database (called MetXBioDB) containing detailed annotations of experimentally confirmed metabolic reactions, (2) a reaction knowledgebase containing generic biotransformation rules, preference rules, and other constraints for metabolism prediction, and (3) a reasoning engine that implements both generic and transformer-specific algorithms for metabolite prediction and selection. The BMPT machine learning system uses a set of random forest and ensemble prediction models for the prediction of CYP450 substrate selectivity, and for the Phase II filtering of molecules. BioTransformer’s Metabolite Identification Tool builds on the BMPT to identify specific metabolites using mass spectrometry (MS) data, namely accurate mass or chemical formula information.

**Fig. 3:**
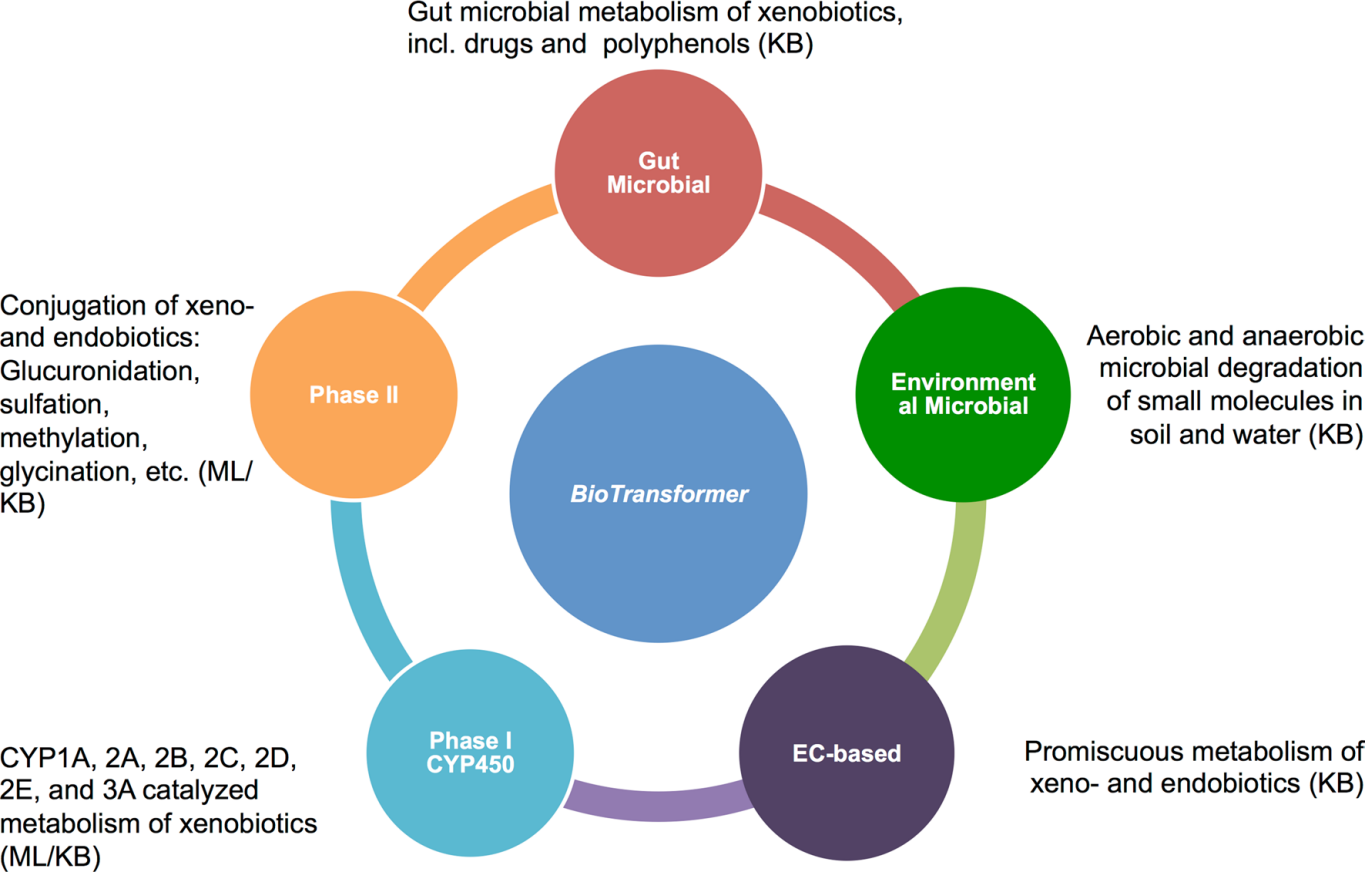
An overview of BioTransformer’s five metabolism prediction modules, the Enzyme Commission based (EC-based), Cyochrome P450 (CYP450), phase II, human gut microbial, and environmental biotransformer modules. *ML* machine learning-based, *KB* knowledge-based, *CYP* cytochrome (P450)

In this section, we describe the structure, content, and implementation of MetXBioDB, the knowledgebase, the reasoning engine, the CYP450 metabolism and Phase II prediction systems, and the metabolite identification tool. Figure 3 gives a brief overview of each “transformer” module, their tasks, and the type of prediction approach they employ. Additional file 2: Figure S1 illustrates the design workflow for the aforementioned BioTransformer components. Finally, we will describe BioTransformer’s workflow, and the RESTful web service.

#### MetXBioDB: a database of metabolites and experimentally confirmed biotransformations and biodegradations

MetXBioDB is a database that consists of a manually curated collection of > 2000 experimentally confirmed biotransformations derived from the literature. It was developed to help with: (1) the design of biotransformation rules, (2) the training and validation of machine learning metabolism prediction models, and (3) the design of preference rules. Each biotransformation in MetXBioDB includes a starting reactant (structure and identifiers), a reaction product (structure and identifiers), the name or type of the enzyme catalyzing the biotransformation, the type of reaction, and one or more citations. For the purposes of this paper, a reactant is defined as a small molecule that binds to a specific enzyme and undergoes a metabolic transformation catalyzed by that enzyme. A biotransformation describes the chemical conversion or molecular transformation of a reactant to one or more products by a specific enzyme (or enzyme class) through a defined chemical reaction. Cytochrome P450 enzymes (CYP450s) are responsible for > 90% of phase I oxidative reactions and > 75% of drug metabolism [58], while UDP-glucuronosyltransferases (UGTs) and sulfotransferases (SULTs) are responsible for the phase II metabolism of most xenobiotics [59, 60] In the gut microbiota, enzymatic reactions are mostly reductive, and are carried out by anaerobic bacteria due to the very low concentration of oxygen.

The “starting” reactants in the current version (version 1.0) of MetXBioDB primarily consist of xenobiotics such as drugs, pesticides, toxins and phytochemicals. The database also includes a small number of sterol lipids and a selected set of mammalian primary metabolites. In assembling MetXBioDB we gathered reaction data from the existing literature (> 100 references) along with data downloaded from publicly available databases such as DrugBank [38], PharmGKB [61], XMETDB [62], and SuperCYP [63]. These databases list over 1000 enzyme-substrate associations for the major CY4P50s and UDP-glucuronosyltransferases (UGTs). Along with published scientific reports, PhenolExplorer [64] and PhytoHub [40] were also used to compile information about the metabolism of polyphenolic compounds in the gut.

The data curation process consisted of three phases including: (1) the collection of biotransformation data, (2) the creation and annotation of biotransformation objects and, (3) data validation. This process was conducted collaboratively with a small team of chemistry experts. A detailed description of the data collection and curation process is provided in the Additional file 2. Additional file 2: Figure S2 illustrates one entry in MetXBioDB, corresponding to the oxidation of acetaminophen to *N*-acetyl-*p*-benzoquinone (NAPQI). Overall, MetXBioDB contains > 2000 biotransformations, which include the cytochrome P450-catalyzed phase I reactions of ~ 800 unique starting reactants (and > 1500 reaction products), the phase II reactions of > 500 unique starting reactants (and > 600 reaction products) and human gut microbial metabolism of > 50 unique polyphenolic compounds.

#### The reaction knowledgebase

BioTransformer’s reaction knowledgebase contains chemical reaction descriptions and rules encoded by SMARTS [65] and SMIRKS [66] strings that are used by the reasoning engine to make biotransformation predictions. This knowledgebase encodes information about, and contains mapping data between, five different concepts: (1) the biosystem, (2) the metabolic enzyme, (3) the metabolic reaction, (4) the metabolic pathway, and (5) the chemical class (as determined by ClassyFire [67]). These concepts are defined as follows:

1. A biosystem is a living organism or a community of living organisms within which the biotransformation reactions can occur. Currently, the implemented biosystems are: (a) the human organism, (b) the human gut microbiome, and (c) the environmental microbiome.
2. A metabolic enzyme is an enzyme that catalyzes or accelerates a metabolic reaction.
3. A metabolic reaction is a chemical reaction that modifies the structure of a molecule, leading to the generation of one or more products.
4. A metabolic pathway is a linked series of chemical reactions that occur in a specific order in the cell or within an organism. A metabolic pathway is organism-specific as an enzyme can be expressed by some organisms but not by others.
5. A chemical class refers to a group of chemicals that share a common structural feature or a group thereof as defined using ClassyFire [67].

The interrelationships between the different concepts are illustrated in Additional file 2: Figure S3. The construction of the reaction knowledgebase required data acquisition and aggregation from several sources, including the information captured in MetXBioDB. Additional reaction information was gathered from resources such as the SIB Bioinformatics Resource Portal (ExPASy) [68], the BRENDA enzyme database [69], various Cyc databases [70], the UniProt knowledgebase (UniProtKB) [71], the KEGG database [47], and enzyme nomenclature information provided by the International Union of Biochemistry and Molecular Biology (IUBMB) [49]. The collected data was used to: (1) design, test, and validate generic reaction/transformation rules, (2) add constraints and rules that would be used by the reasoning engine, and (3) map entities from different concepts. An example of the type of concept mapping done for the reaction knowledgebase is given here: phosphatidylcholines are a chemical class, the glycerophospholipid metabolism pathway is a metabolic pathway, a human is a biosystem, therefore phosphatidylcholines are mapped to the glycerophospholipid metabolism pathway in humans.

Based on the information gathered from the various resources, 423 associations could be established between the reaction knowledgebase’s enzymes and reactions. Priority was given to enzymes with wide substrate specificity such as the arylamine N-acetyltransferase (EC 2.3.1.5), as the aim was to predict the metabolism of small molecules partly based on generic biotransformation rules. Exceptions included, for example, serine palmitoyltransferase (EC 2.3.1.50), which is a specific enzyme that provides the sphingoid base 3-dehydrosphinganine needed for the biosynthesis of sphingolipids. All biotransformation rules in the knowledgebase were encoded in the SMIRKS language [66]. For each biotransformation rule, one or more structural constraints (e.g. the known enzyme substrates are restricted short-chain fatty acyl chains) were encoded separately, either in the SMARTS language [65] or programmatically (by combining several rules based on the structural constraints and/or physicochemical properties). The reaction SMIRKS descriptions, and SMARTS-encoded constraints are freely available at https://bitbucket.org/djoumbou/biotransformerjar/.

The separate design of structural constraints was necessary for several reasons. First, structural constraints can sometimes be difficult or impossible to fully encode using the SMIRKS language alone, due to its limited expressivity. Second, the juxtaposition of constraints within a SMIRKS pattern can make it difficult to understand, and cumbersome to update. A typical reaction scheme encoded in the reaction knowledgebase is shown in Additional file 2: Figure S4. Once a reaction was encoded, several tests were performed to assess its correctness by applying the reaction to known substrates as well as to known non-substrates (i.e. chemicals that were known not to satisfy the various constraints). If the reaction passed all the tests, it was added to the database; if it failed, the reaction schema was subject to one or more iterations and tests until validated.

Some of the encoded reactions in the reaction knowledgebase apply to a very specific set of chemicals, and can be used to accurately predict the metabolism of compounds belonging to those classes. Such examples include the aforementioned conversion of diacyl-sn-glycero-3-phosphoethanolamines to diacyl-sn-glycero-3-phosphoserines, and the metabolism of several classes of lipids, which are known to follow classic primary metabolic pathways. Other reactions are so generic or non-specific that they would lead to a high number of false predictions if applied blindly. Some examples of highly non-specific reactions include aliphatic hydroxylation, *N*-dealkylation, and glucuronidation, among many others. These reactions are catalyzed by enzymes that have broad substrate specificity, such as CYP450s and UGTs. To handle these situations, new reaction subtypes and constraints were defined, which focused on a specific subclass of compounds that fulfilled a defined set of structural constraints. The resulting manually generated rules were then subject to further testing and validation. An example of such a reaction/rule is the *N*-dealkylation of alicyclic tertiary amines catalyzed by CYP3A4, a well-studied bioactivation pathway of cyclic amines [72].

In addition to the core knowledge provided by textbooks, online databases and journal articles, the design of biotransformation rules for the reaction knowledgebase often required additional investigation. One approach consisted of selecting compounds (from MetXBioDB) that triggered a given reaction and labeling them based on whether their expected metabolites were reported or not. Further analysis of these reaction sets often suggested new reaction schemes or the addition of new constraints to existing reaction schemes. A similar process was previously used to generate > 300 biotransformation rules for the prediction of environmental microbial metabolism [33, 73]. These rules were also encoded, tested, and added to BioTransformer’s reaction knowledgebase. Overall, a total of 797 biotransformation rules were encoded, tested, and eventually added to the reaction knowledgebase.

In addition to identifying the mechanisms involved in various metabolic reactions, and encoding of biotransformation rules, another challenge to building the reaction knowledgebase was determining the prioritization needed for specific metabolic reactions. For any compound that triggers several competing reactions, certain reactions are more likely to occur than others. Therefore the metabolites resulting from these preferred reactions are more likely to be observed. Given a pair of metabolic reactions, a common approach to define precedence rules involves a detailed analysis of common putative and observed metabolites via NMR or mass spectrometry [73]. Another approach involves using NMR or mass spectrometry to perform time-course monitoring of biotransformations in order to elucidate the preferred metabolic pathways [74]. In this work, our construction of precedence rules between pairs of reactions was mostly based on data acquired from previously reported scientific studies, as well as observations published in previous studies.

For instance, when absorbed in the intestine, polyphenolic compounds must be deconjugated (via glycosidases or carboxylesterases) before undergoing any transformation [75, 76] Recently, Burapan et al. [74] investigated the regioselectivity of *O*-demethylation of polyphenols by the human gut bacterium *Blautia* Sp. MRG-PMF1, and concluded that *O*-demethylation of polymethoxyflavones occurs most preferably at the C-7 position, compared to the C-4′ and C-3 positions. Based on these observed patterns, kaempferol 7,4′-dimethyl ether 3-glucoside (see Additional file 1) would more likely undergo *O*-deglycosylation, followed by C-7 *O*-demethylation to give kaempferol 4′-methyl ether (see Additional file 1), which will then undergo further metabolism (Additional file 2: Fig. S5). In total, 190 precedence rules were created for 49 unique biotransformation rules that were encoded for the human and/or human gut microbial biosystems. These precedence rules were created based on observations reported in scientific articles, or personal communication with experts. In addition, 1960 precedence rules for 195 unique biotransformation rules were adopted from the EAWAG-BBD/PPS system (environmental microbial metabolism). Not all reaction schemes in the reaction knowledgebase are fully specified. For instance, because relatively little is known about the biology and enzymology of the human gut microflora, a large number of encoded biotransformation rules were either assigned to an enzyme superfamily or to an “unspecified enzyme”. For the Knowledgebase’s collection of environmental microbial reactions, the biotransformation rules were assigned to a single “unspecified enzyme”, as they are often consensus rules designed by combining patterns of reactions catalyzed by several enzymes. Overall, upon validation of the reactions and the addition of constraints, 1716 enzyme-based reaction associations were created.

The next step in constructing the reaction knowledgebase consisted of associating enzymes with metabolic pathways, and the corresponding biosystems. This step is very important for several reasons. First, many metabolic pathways are organism-dependent as different organisms express different enzymes or transporters (Additional file 2: Figure S3). Thus, as illustrated in Additional file 2: Figure S3, the metabolic route linking a compound to a metabolite could vary between organisms. While sphingomyelins can be directly converted into ceramide-1-phosphates in *Aspergillus Flavus*, humans must convert sphingomyelins into ceramides first, which are then transformed into ceramide-1-phosphates. Second, the mapping also allows one to encode more constraints and exclusion rules for certain types of compounds. For instance, glycerophospholipids are transformed solely within the glycerophospholipid metabolism pathway, and do not undergo CYP450- or UGT-catalyzed metabolism. In total, seven metabolic pathways were created, 84 enzyme-pathway associations, and nine chemical class-pathway associations were created for the human biosystem. A summary of the numbers of rules and associations encoded in the reaction knowledgebase are shown in Table 1 for each of the five transformer modules (EC-based, human CYP450, human gut microbial, phase II, and environmental microbial). The biotransformation rules and the list of enzymes cover all six enzyme classes EC1 through EC6 of the Enzyme Nomenclature, as defined by the IUBMB [49], with deeper focus on classes EC1 to EC4. The metabolic pathways are currently limited to lipid metabolism. The annotation and mapping of all enzymes, metabolic reactions, biosystems, metabolic pathways, and chemical classes are freely available at https://bitbucket.org/djoumbou/biotransformerjar/.

**Table 1:** Statistics for each of the five transformer modules: (1) EC-based module (Enzyme Commission-based metabolism); (2) CYP450 module (Cytochrome P450 metabolism); (3) human gut microbial module (Human gut microbial metabolism); (4) Phase II module (Phase II metabolism), and (5) environmental microbial module (Environmental microbial degradation)

	Number of enzymes	Number of biotransformation rules	Number of enzyme-rule associations	Number of covered biosystems
EC-based (ecbased)	285	408	459	2
CYP450 (cyp450)	9	163	712	1
Human gut microbial (hgut)	53	201	204	2
Phase II (phaseII)	9	74	81	2
Environmental microbial (envmicro)	1	301	301	1

The codes/abbreviations in the first column are the names of options used programmatically to specify the module of interest

#### The reasoning engine

The BMPT’s Reasoning Engine uses the rules in the reaction knowledgebase to select the most likely of all applicable metabolic biotransformations or pathways. In general, two types of reasoning are used for the selection and ranking of predicted metabolites: absolute reasoning, and relative reasoning [77]. Absolute reasoning solely focuses on the likelihood of a biotransformation to occur, and is used to select the biotransformations with an occurrence ratio above a given threshold. Examples of biotransformation software using absolute reasoning include SyGMA and Meteor Nexus. Relative reasoning evaluates the comparative likelihood between two independent but competing reactions (e.g. flavone 7-*O*-demethylation is more likely to occur than flavone 4′-*O*-demethylation [74]. Examples of computational tools using relative reasoning include Meteor Nexus and the EAWAG-BBD/PPS system. Both absolute and relative reasoning have been implemented. However, in the current version of BioTransformer all reaction patterns have been assigned the same likelihood. The computation of more accurate reaction-specific scores requires a larger set of data, which is still being assembled and tested. We aim to provide more accurate reaction scores in a future version of BioTransformer that will be released in 2019.

Besides qualitative attributes (e.g. chemical class), reasoning engines often also use quantitative attributes (e.g. mass, LogP) to guide their predictions. BioTransformer’s reasoning engine uses both types of attributes. While chemical classification can help to select the most likely biotransformations or discard the unlikely ones, quantitative attributes such as the mass and LogP are used to predict the substrate specificity for various enzymes, or whether a known molecule is hydrophilic enough to be conjugated/eliminated. For the prediction of enzyme-substrate specificity, the current version of BioTransformer focuses on nine of the most “active” or best-studied CYP450 enzymes (CYP1A2, CYP2A6, CYP2B6, CYP2C8, CYP2C9, CYP2C18, CYP2D6, CYP2E1, and CYP3A4). The prediction of their specificity toward a given substrate is made by CypReact [57] a machine learning software tool for CYP450 reaction prediction that was recently developed by our team. To predict whether a compound is hydrophilic enough for conjugation/elimination, BioTransformer uses its internal, machine learning Phase II filter that use structural fingerprints, and physicochemical properties (e.g. LogP, mass) to select likely Phase II candidates. CypReact, and the Phase II filter will be briefly described in the next section.

With the reaction knowledgebase and the machine learning tools in hand, the Reasoning Engine was implemented programmatically for each of the five different transformer modules. The rationale behind this design was to have independent transformer modules that could be used separately. This way, one could focus on a specific type of metabolism (e.g. CYP450-catalyzed metabolism) or a specific type of biosystem (human). Among the five transformer modules, three rely solely on the application of rules and constraints from the reaction knowledgebase. These three are the EC-based transformer, the human gut transformer and the environmental transformer. The cytochrome P450 (Phase I) transformer, which focuses on the metabolism of small molecules mediated by CYP450 enzymes, and the Phase II transformer, are the only transformers that implement a machine learning approach in combination with a knowledge-based approach. In addition to the five transformer modules, the Reasoning Engine is used by a combined human “super transformer”, which aims at simulating the metabolism of small molecules in humans (including the human gut), from their absorption to their excretion.

#### The CYP450 metabolism prediction system

Cytochrome P450 enzymes (CYP450s) constitute a superfamily of heme proteins, with over 50 isozymes identified in humans [78]. They are predominantly found in the liver, but also occur in other organs such as the lungs, the kidneys, the gut wall, and the small intestine. CYP450s are the major oxidative enzymes in the human body, and are responsible for the metabolism of a large number of compounds. Nine specific CYP450s have been identified as responsible for most of the Phase I metabolism of xenobiotics (e.g. drugs, food additives, and environmental contaminants) and a small number of endogenous compounds. These include the CYP1A2, CYP2A6, CYP2B6, CYP2C8, CYP2C9, CYP2C18, CYP2D6, CYP2E1, and CYP3A4 isozymes. Because of their broad substrate specificity, a special CYP450-reactant specificity prediction was implemented, in order to predict metabolites for the more likely reactants. The enzyme-specificity is assessed by a program called CypReact [57].

CypReact is a software tool that uses a machine learning approach to predict whether a small molecule reacts with any of the nine major CYP450 isozymes. CypReact uses a random forest model for each of seven isozymes (CYP1A2, CYP2A6, CYP2B6, CYP2C8, CYP2C19, CYP2E1, CYP3A4), and ensemble models for two isozymes (CYP2C9, CYP2D6). Each of the models uses a set of physicochemical properties and structural features of a molecule for substrate specificity prediction. The substructure fingerprints were partly developed by including a subset of SMARTS pattern definitions from ClassyFire [67], and a set of SMARTS patterns known to trigger CYP450-catalyzed metabolism (e.g. p-substituted phenols, or *N*-substituted piperazine). These fingerprints encode other pattern definitions for key functional groups and structural features relevant to CYP450-catalyzed metabolism, which were obtained through data mining. In addition, the corresponding PubChem fingerprint [79] and the MACCS fingerprint [80] were added. Feature selection, and parameter optimization, cost-sensitive learning, and cross-validation based evaluation were performed to design highly accurate models for each CYP450 model. Empirical results show that CypReact’s classifiers can achieve a very high performance, with AUROC scores ranging between 83% and 92%. Moreover, they were shown to significantly outperform SmartCyp [24], and ADMET Predictor [29]. For a more detailed description about the list of fingerprint generation, training process, and resulting models, the user is referred to the CypReact paper [57]. In addition to the nine models, CypReact also uses a heuristic approach to filter candidates that are known to be out of scope for CYP450 mediated metabolism, based on their chemical structure and/or physicochemical properties. These include inorganic compounds, and several classes of glycero- and glycerophospholipids, among others. CypReact is freely available at https://bitbucket.org/Leon_Ti/cypreact/.

Given any small molecule, the CYP450 transformer uses CypReact to predict which of the nine CYP450s is likely to metabolize the molecule. Subsequently, it implements the constraints and biotransformation rules encoded within the reaction knowledgebase to predict the structures of the resulting metabolites. As for any other transformer module, the user can vary the parameters, including the number of transformation steps, and whether to use certain precedence rules.

#### The Phase II metabolism prediction system

Phase I reactions tend to render the lipophilic xenobiotics more reactive by adding or modifying functional groups, such as an amino-, hydroxyl-, or carboxyl group. Some examples of Phase I reactions include aliphatic hydroxylation, and epoxide hydrolysis. In Phase II, the more reactive metabolites are conjugated to cofactors, making them less toxic, more hydrophilic, and thus easier to eliminate. Some of the more common Phase II reactions include the conjugation of xenobiotics to glucuronic acid (glucuronidation), sulphate (sulfation), a methyl group (methylation), an N-acetyl group (N-acetylation), glutathione, taurine, and glycine. These reactions are catalyzed by the families of UDP-glucuronosyltransferases (UDP-GTs), sulfotransferases (SULTs), methyltransferases (MTs), *N*-acetyltransferases (NATs), glutathione transferases (GSTs), bile acid-CoA:amino acid *N*-acyltransferase (BACATs), and glycine transferases (GTs), respectively. While the presence of adequate attachment and functional groups is required for conjugation, the lipophilicity of a molecule is also significantly influenced by its shape, mass, and functional group composition, among other parameters. Therefore, a simple structure-based chemical classification would not be enough to predict whether a candidate molecule is suitable for Phase II. In order to provide an accurate prediction, we designed the Phase II Filter (P2F).

The Phase II Filter was designed as a simple machine learning model that takes physicochemical properties as well as structural features of a molecule to predict whether it is ready for Phase II metabolism. A compound is predicted as Phase II ‘ready’ if it can undergo one or more transformations catalyzed by any of the six aforementioned enzyme families. In contrast to CypReact, which combines nine independent predictors (one for each CYP450 isozyme), the P2F consists of a single machine learning model.

Because of the broad specificity of the aforementioned Phase II enzymes, especially UPD-GTs and SULTs, it was important to collect as structurally diverse a set as possible. Selected compounds included xenobiotics (e.g. pharmaceuticals, pesticides, food additives, toxins, phytochemicals), as well as endobiotics (e.g. steroids, bile acids, amino acids). A total of 1113 compounds were collected from several databases, including MetXBioDB, PubChem [79], BRENDA [69], and the Cyc databases [70], as well as the scientific literature. The training set contained 807 Phase II substrates, and 306 Phase II non-substrates. When unavailable from any of the sources, the structure of a compound was generated using ChemAxon’s MarvinSketch v.17.2.27.0 [81]. Standardization operations (e.g. removal of salts, and 3D structure generation) were also performed. Certain classes of compounds, such as glycerolipids, are known not to undergo conjugation by any of the Phase II enzymes. Since these compounds could be pre-filtered using a simple structure search, they were not included in the training set. Furthermore, compounds that do not contain adequate reaction sites (i.e. functional groups that could be attacked by Phase II enzymes) were not included. This is because such compounds could be easily filtered by structural pattern matching.

After the collection and standardization of our training set, a total of 32 molecular descriptors were calculated for each of the 1113 molecules. These included nine constitutional descriptors and molecular properties (e.g. the number of H-bonds, the mass, and the AlgoP), as well as 23 structural features, such as amine groups (SMARTS = “[NX3+0,NX4+;!$([N]~[!#6]);!$([N]*~[#7,#8,#15,#16])]”), and carboxyl groups (SMARTS = “[#8;A;X2H1,X1-][#6]([#6,#1;A])=O”). The molecular descriptors were all computed with the CDK library. The structural features are represented as binary features in a custom chemical fingerprint to encode their absence (0) or presence [1] in the query molecule. A list of structural features and physicochemical parameters is available in Additional file 3: Table S1.

Feature selection was performed to select a set consisting of the features that are most significant in explaining the training data. This not only accelerated the training/prediction process but also reduced the likelihood of overfitting. Feature selection was performed on the Waikato Environment for Knowledge Analysis (WEKA) [82] using the information gain criteria and a ranker. Overall, 25 physicochemical properties and structural features were selected to build and evaluate several models (evaluated by 10-fold cross validation) using several different machine learning algorithms (i.e. decision trees, random forest, and naïve Bayes). Upon comparative evaluation of the F-1 measure and ROC area, a random forest model was selected as the best predictor. The model achieved a weighted average F1-measure of 0.88, and a weighted average ROC area of 0.94.

Our training was limited to compounds possessing necessary structural motifs (e.g. functional groups) that are targeted by the aforementioned Phase II enzyme classes for conjugation. A number of chemical classes, including ether lipids, glycerolipids, and glycerophospholipids, sphingolipids, and acyl-CoA conjugates were excluded from the training set, as such compounds are known either not to be transformed by any of the seven Phase II enzyme classes, or to be conjugated following a very specific metabolic pathway. In the latter case, the chemical class-to-pathway associations encoded in BioTransformer’s reaction knowledgebase would allow for a more accurate biotransformation prediction, if applicable. For these reasons, a simple rule-based filtering module was implemented to eliminate the most trivial non-candidates, before applying the trained model. The rule-based module excludes compounds from the five aforementioned chemical classes. Moreover, only compounds with a molecular weight lower than or equal to 900 Da (selected based on extensive internal analysis of our collected data), and containing a limited set of 64 different structural motifs (see Additional file 3: Table S2) are then passed to the machine learning filtering module.

#### The BioTransformer metabolite identification tool

Metabolite identification is one of the main tasks of untargeted metabolomics. The aim of untargeted metabolomics is to analyze biofluids (e.g. urine, blood) from an organ or organism and to attempt to identify novel metabolites that are characteristic of that organism’s response to an exposure to a chemical or other stimuli. Mass spectrometry (MS) is one of several analytical approaches used to perform this task. When coupled with (gas or liquid) chromatography, a mass spectrometer produces a set of spectra that contain features (e.g. mass-to-charge ratios, peak intensities, calculated molecular formulas) characteristic of metabolites or fragments thereof. While spectral searching is a method commonly used to identify metabolites, the lack of reference spectra for many metabolites is a bottleneck in rapid and accurate compound identification. Therefore, the comparison of spectral features (e.g. mass, molecular formula) obtained from mass spectra with those obtained from metabolism prediction data could help to putatively identify known or unknown metabolites and validate predictions.

The BioTransformer metabolite identification tool (BMIT) is an additional module within BioTransformer that is designed to assist users in metabolite identification. It relies on the BMPT to find compounds of a specific mass (within a user-specified threshold) or chemical formula that are generated upon single- or multistep metabolism of a given parent molecule. BMIT takes the chemical structure of the starting molecule as input, as well as a list of neutral chemical masses or molecular formulas for the metabolites to be identified. BMIT is implemented to only support metabolite identification using the *allHuman* and *superbio* options (Human + Human Gut Microbiome), or the *envmicro* option (Environmental Microbiome). The search for metabolites is applied iteratively at each step, and stops when at least one metabolite has been identified for each given mass (± a mass tolerance) or given chemical formula or when the maximal number of steps has been reached. If applicable, the BMIT returns each matching metabolite, including its structure, its chemical formula, its molecular mass, and a pathway leading to it, starting from the query compound. The results are saved in a single SDF file in which each pathway is stored as an ordered list of chemical reactions (with reaction name, and a list of catalyzing enzymes).

#### BioTransformer’s input and workflow

BioTransformer was implemented in the Java programming language, and can be used as a command-line tool (on Linux, Mac OSX, and Windows) to perform metabolism prediction and metabolite identification of small molecules. Beside CypReact, described earlier, BioTransformer uses two other open source tools, namely the Chemistry Development Kit (CDK) [83] and the AMBIT library [84]. The CDK programming library is used for several operations, including the calculation of physicochemical properties, the execution of superstructure search operations, and the handling of chemical structures, among others. The AMBIT library is used for the application of biotransformation rules and structure generation.

**Fig. 4:**
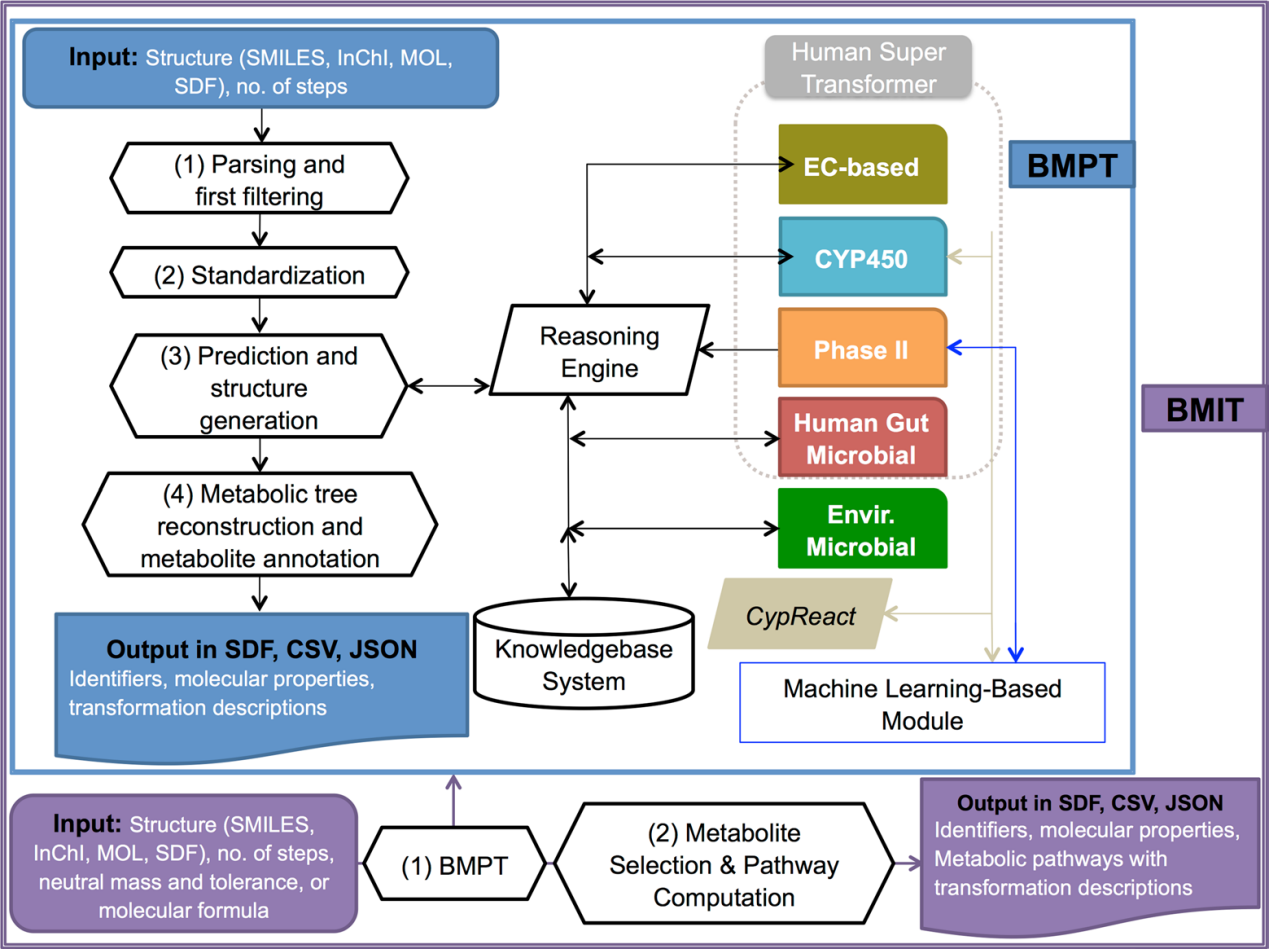
Workflow of BioTransformer’s metabolism prediction and metabolite identification tools. The BioTransformer metabolite prediction tool (BMPT) is used solely for metabolism prediction. For metabolite identification tasks, the BioTransformer metabolite identification tool (BMIT) makes use of those predictions to suggest putative metabolites of a compound that have a given neutral mass or molecular formula

The BioTransformer metabolism prediction tool’s workflow is illustrated in Fig. 4. As can be seen in this diagram, BioTransformer accepts molecules either in SMILES (single molecule), InChI (single molecule), MOL (single molecule), or SDF (single or multiple compounds) format as input. Each molecule must be an organic molecule and it must not be a mixture or a salt. Once the input is parsed, the structures are subjected to chemical validation and standardization. The standardization process consists of removing charges from functional groups (with some exceptions, such as nitro groups), checking and validating bond types and adding explicit hydrogen atoms. Subsequently, BioTransformer predicts the biotransformations and the resulting metabolite structures for each query molecule separately (see Additional file 2: Fig. S6). In some cases, the structural representation of a molecule upon standardization can differ slightly from the original one. Therefore, we encourage users to provide identifiers (e.g. custom labels, names, etc.) in addition to the structural representation. This is even more relevant when a BioTransformer prediction is used as part of an automated workflow.

Each prediction must be run in the single module mode, where the user selects one of the five transformer modules (CYP450, EC-based, phase II, gut microbial, or environmental microbial). The Biotransformer options used to specify the modules are *cyp450* (CYP450 metabolism module), *ecbased* (EC-based metabolism module), *phaseII* (Phase II metabolism module), *hgut* (Human gut microbial degradation module), and *envmicro* (Environment microbial degradation module). Alternately, a human “super transformer” has been implemented to mimic the metabolism of small molecules in the human “superorganism”, which also includes the gut microbiota. This super transformer integrates the CYP450, EC-based, phase II, gut microbial transformers and covers a number of different reaction types, including hydrolysis, oxidation and reduction, and conjugation. The “super transformer” provides two options: (1) *allHuman*, which uses all four human-related transformers at each step of the prediction, or; (2) *superbio*, which uses all the human-related biotransformers in an ordered sequence of up to 12 steps, starting with the hydrolysis of the query molecule (if applicable), and ending with the conjugation of its metabolites.

After the metabolite prediction step is completed, the structures and biotransformations are annotated (Fig. 4). Based on the information from the predicted biotransformation(s), BioTransformer builds a metabolic tree by associating each metabolite with its parent(s). Moreover, each predicted metabolite is annotated with additional information that provides structural identification, reports its physicochemical properties, and an explanation of its origin or provenance. The data includes: (1) three chemical identifiers (metabolite ID, InChI, InChI Key), (2) the molecular formula, (3) the monoisotopic mass, (4) the reaction type leading to the metabolite, (5) the biosystem that generated the molecule, (6) the parent compound identifiers (BioTransformer ID, InChIKey), (7) the parent monoisotopic mass, (8) the metabolite’s and parent’s AlogP, as well as (9) the metabolite’s and parent’s synonyms. The results are returned in a SDF or CSV file that contains the structure and annotation of the predicted metabolites. The returned information can be used separately to analyze metabolic pathways. It can also be used to compute neutral losses for MS-based analyses that can be used to experimentally detect each biotransformation.

BioTransformer’s metabolite identification tool (BMIT) builds from the metabolism prediction tool (BMPT). Given a starting molecule, a set of molecular masses and a mass tolerance threshold (in Da) or simply a set of molecular formulas, BMIT identifies potential metabolites for each valid mass or molecular formula, via single or multi-step metabolism, depending on the user input. For mass-based searches, the default number of steps, and mass tolerance are set to one, and 0.01, respectively. The user can select to explore the human and human gut microbiome environments (with the *allHuman* and *superbio* options), or the environmental microbial metabolism (with the option “*env*”). A metabolic pathway linking the starting structure and each of the metabolites is returned, based on the metabolic tree obtained upon metabolism prediction. Metadata include the structures, identifiers, reaction types, and enzymes.

#### The BioTransformer web service

The BioTransformer software package can be used as a command line tool or as a Java library. In order to further facilitate access to this tool, a RESTful web service was built using the JRuby on Rails framework. The BioTransformer web service is freely available at www.biotransformer.ca. The web service allows users to manually or programmatically submit queries, and retrieve the corresponding results using the workflow described in the previous section. In particular, the web service allows users to submit compounds in SMILES, InChI, and SDF formats (Additional file 2: Fig. S7). Query results can be returned as JSON, SDF, and CSV documents (Additional file 2: Fig. S8). Moreover, the web server provides information about each previously predicted single-step metabolic transformation of the compound, including the corresponding biosystem, reaction type, metabolizing enzymes, and transformation products. The web application offers several advantages compared to the command-line tool, namely: (1) it is easier to use than the stand-alone program; (2) users need not be programmers or need to install a local program to run the web service; (3) several queries can be processed simultaneously; (4) the computation is faster, as previous prediction results are saved in a database to facilitate more rapid retrieval; and (5) metabolite prediction and identification data can be accessed manually or programmatically and downloaded in several formats. While the command-line executable does not benefit from the database of computed metabolites, it also does provide some advantages, namely: [1] it allows users to submit large sets of compounds; [2] it does not rely on an Internet connection, and; [3] queries are executed immediately and not put in a queue.

#### Evaluation of BioTransformer’s metabolism prediction and metabolite identification capabilities

In order to evaluate the performance of BioTransformer, we performed a comparative analysis with two popular in silico metabolism prediction tools, namely Meteor Nexus [26], and ADMET Predictor [29]. Moreover, we evaluated BioTransformer’s ability to replicate environmental microbial metabolism prediction from the EAWAG BDD/PPS system [33, 34, 73]. We also tested BioTransformer’s ability to predict comprehensive human and gut metabolism of small molecules. Building on BioTransformer’s metabolism prediction ability, we also tested its metabolite identification capabilities with the BMIT module. For each of the tests, BioTransformer was run on a 2.7 GHz Intel Core i5 MacOSX with 16 GB (1867 MHz DDR3) of memory. The procedures and results are presented in the Results section.

## Results

### Comparative evaluation of BioTransformer and Meteor Nexus in the prediction of human single-step metabolism of small molecules

The first test involved a comparative assessment of the performance of BioTransformer and Meteor Nexus (v.3.0.1) [26] in predicting single-step human metabolism of 40 pharmaceuticals and pesticides, randomly selected from DrugBank [38] and T3DB [42]. This test set was limited to these compound classes because Meteor Nexus’ biotransformation dictionary and associated rule bases are specifically limited to pharmaceuticals and pesticides. Both BioTransformer and Meteor Nexus were set to use absolute/relative reasoning to prioritize the most likely biotransformations. In contrast to BioTransformer, Meteor Nexus clearly defines several levels of reasoning that express different levels of confidence. Therefore, Meteor Nexus’ predictions were computed for each of the equivocal (EQUI), plausible (PLAU), and probable (PRO) levels of confidence. For each compound, the BioTransformer’s predictions were evaluated against a Meteor Nexus prediction obtained at each of the three confidence levels. The assessment was performed by comparing the precision (i.e. the fraction of true metabolites among the predicted ones) and recall (i.e. the fraction of true metabolites that were predicted over the total number of true metabolites) for each setting. For details about the evaluation, see Additional files 2 and 4.

**Fig. 5:**
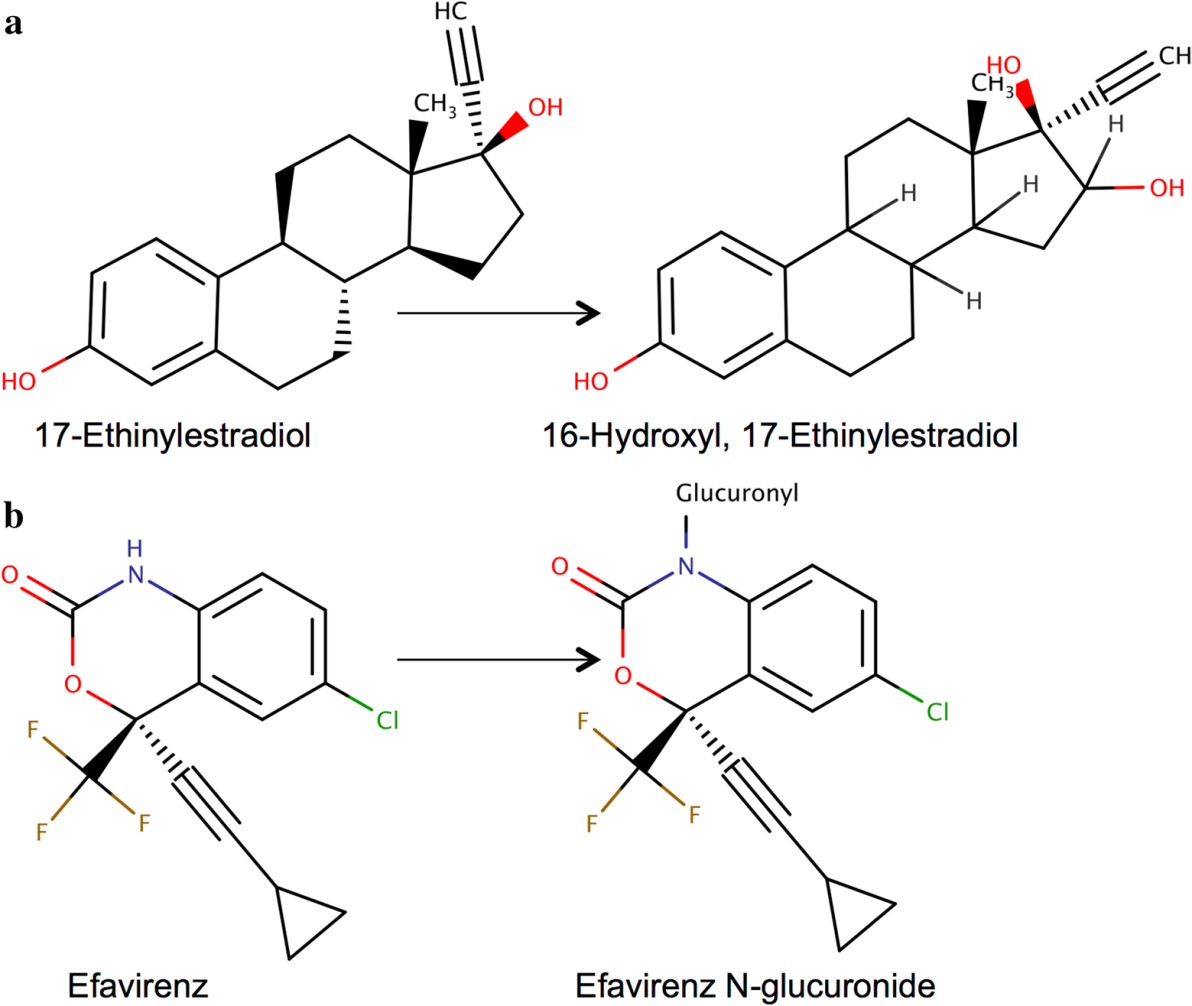
Examples of predicted metabolites: **a** 16-hydroxy-17-ethinylestradiol, a reported metabolite of 17-Ethinylestradiol was predicted by BioTransformer only. **b** Efavirenz, *N*-glucuronide, a reported metabolite of Efavirenz predicted by Meteor Nexus only

BioTransformer’s average computation time was 3.55 s per compound whereas Meteor Nexus’ average computation time was 2.95 s per compound. A summary of the comparative assessment of BioTransformer and Meteor Nexus (Lhasa Limited, UK) is displayed in Table 2. When compared to the Meteor Nexus predictions obtained at the “Equivocal” level of reasoning, BioTransformer achieved higher precision (49% vs. 35%) and recall (88% vs. 71%). As an illustration, BioTransformer predicted 7 out of 8 true metabolites for 17-Ethinylestradiol, compared to 4, 1, and 0 by Meteor Nexus using its Equivocal, Plausible, and Probable levels of confidence, respectively (Fig. 5). On the other hand, Meteor Nexus predicted 3 out of 3 true metabolites for Efavirenz, compared to only 2 for BioTransformer (Fig. 5). Meteor Nexus achieved higher precision at the “Plausible” (56%) and “Probable” (59%) levels compared to BioTransformer. However, this caused a significant drop of the recall to 45% at the Plausible, and 13% at the Probable levels of confidence, respectively, compared to an 88% recall by BioTransformer (see Table 2).

**Table 2:** Comparative assessment of BioTransformer’s and Meteor Nexus’ predictions of human (not including gut microbiome) single-step metabolism for 40 pharmaceuticals and pesticides

	BioTransformer	Meteor Nexus		
		EQUI	PLAU	PRO
True positives	188	152	96	28
False positives	198	279	74	19
False negatives	26	62	118	186
Total no. of predictions	386	433	170	47
Precision	0.49	0.35	0.56	0.59
Recall	0.88	0.71	0.45	0.13
No. of reported metabolites	224			

The different confidence levels implemented by Meteor Nexus (Lhasa Limited) are: EQUIVOCAL (EQUI), PLAUSIBLE (PLAU), and PROBABLE (PROB)

### Evaluation of BioTransformer’s prediction of human and human gut microbial single-step metabolism of small molecules

**Fig. 6:**
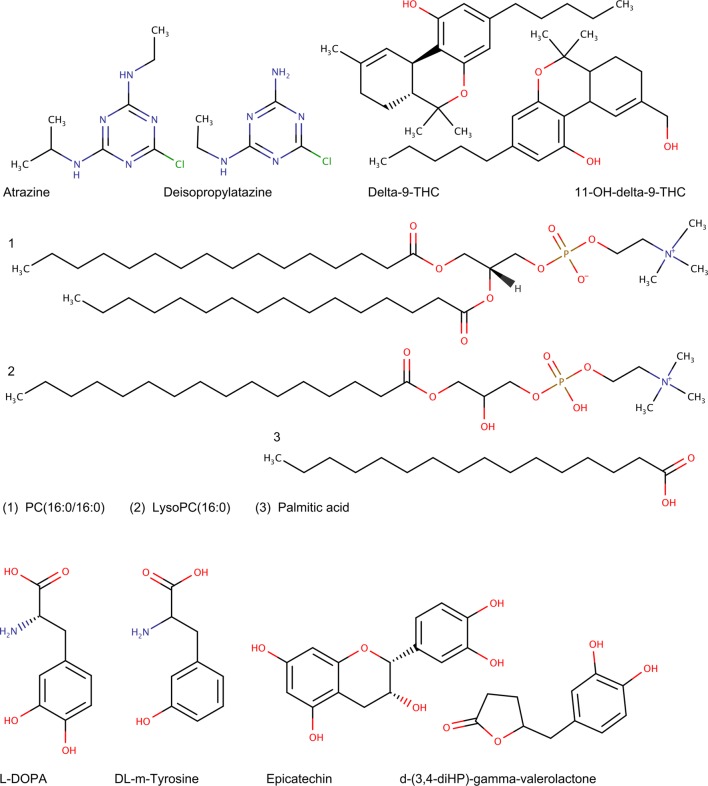
Examples of human (non gut microbial) metabolites predicted by BioTransformer. This figure illustrates the human hepatic metabolites of Atrazine, delta-9-tetrahydrocannbinol (Delta-9-THC), and phosphatidylcholine (16:0/16:0), as well as human gut microbial metabolites of L-DOPA and Epicatechin, correctly predicted by BioTransformer

The second test involved an assessment of BioTransformer’s performance in predicting single-step human and human gut microbial metabolism of 20 well-studied pharmaceuticals, lipids, polyphenols, and other phytochemicals, from the HMDB [2] (none of which was included in the first test set), using the super transformer’s *allHuman* option. This was done to assess BioTransformer’s performance in a task more related to metabolomic or exposomic studies, where the prediction of both endogenous and exogenous metabolites arising from human metabolism is highly desirable. To our knowledge, no commercial or publicly available tool is available that was implemented to perform this kind of diverse metabolite prediction, so no comparison could be done in a fair manner. BioTransformer’s average computation time for this (more comprehensive) analysis was 4.10 s per compound. Overall, BioTransformer achieved a precision of 69% and a recall of 87% (Table 3). Although the set is more chemically diverse, the performance of BioTransformer is actually better than what was achieved for the first test involving pesticides and pharmaceuticals (described above). Examples of predictions by BioTransformer are illustrated in Fig. 6. Details of the evaluation are available in the Additional file 5.

**Table 3:** Evaluation of BioTransformer’s performance in predicting human and human gut microbial metabolism of 20 small molecules

	BioTransformer
True positives	111
False positives	49
False negatives	17
Total no. of predictions	160
Precision	0.69
Recall	0.87
No. of reported metabolites	128

### Comparative Evaluation of BioTransformer and ADMET Predictor in the Prediction of Human Single-step CYP450-mediated Metabolism of Small Molecules

**Fig. 7:**
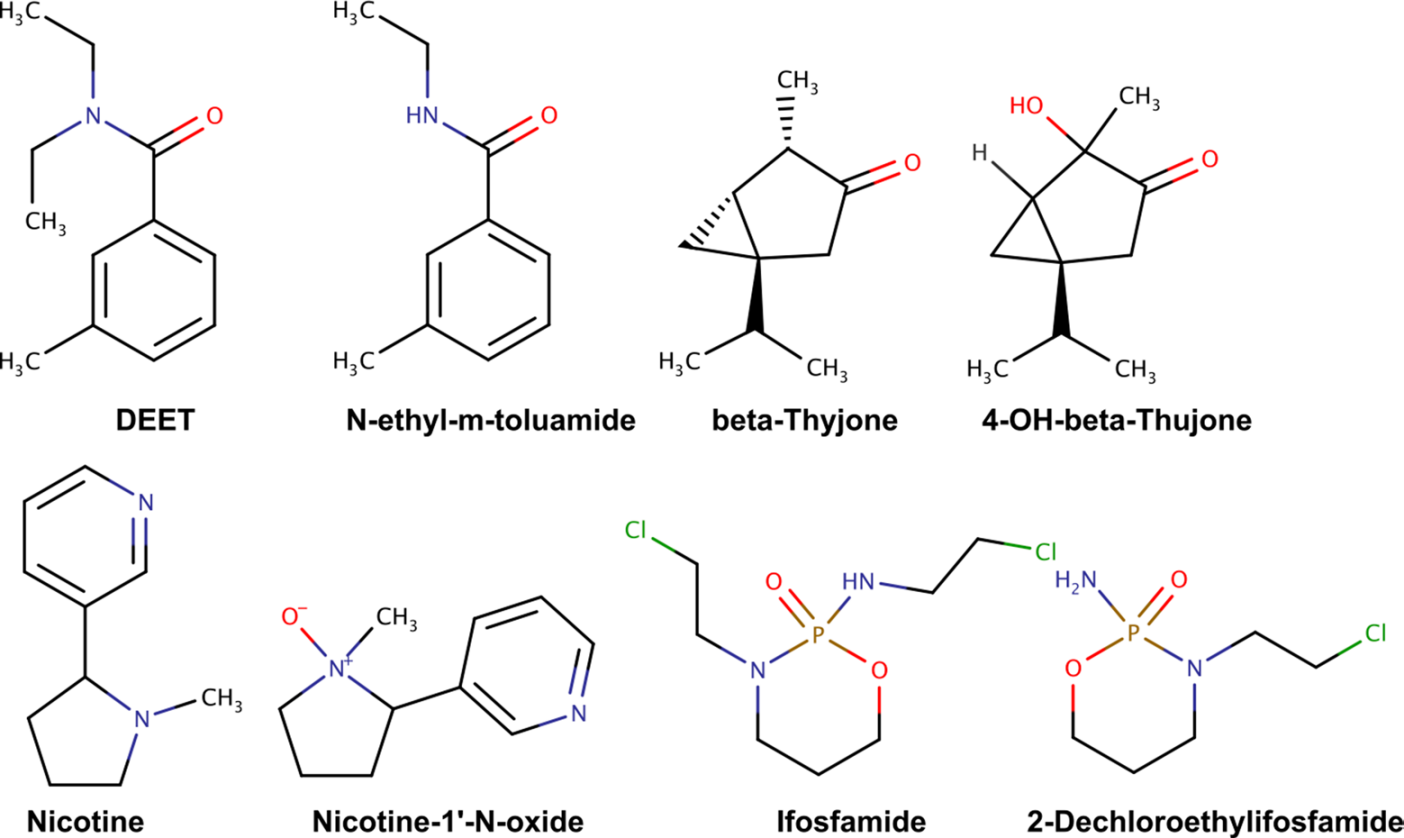
Examples of predicted metabolites as predicted by BioTransformer and ADMET predictor

In our third test, the CYP450-catalyzed single-step metabolism of the 60 aforementioned molecules was predicted using ADMET Predictor (v.8.5.1.1) [29]. ADMET Predictor is a software tool that allows the prediction of sites of metabolism and the resulting metabolites upon CYP450-catalyzed biotransformation. The set of nine CYP450 isoforms supported by ADMET Predictor (1A2, 2A6, 2B6, 2C8, 2C9, 2C19, 2D6, 2E1, and 3A4) is identical to the one covered by BioTransformer CYP450 metabolism prediction tool. The resulting metabolites were compared to those obtained from BioTransformer’s CYP450 metabolism prediction module, and a performance assessment was then carried out (Table 4). BioTransformer’s predictions were computed in an average 2.69 s per compound, while the ADMET Predictor predictions took an average of 0.45 s per compound. BioTransformer and ADMET Predictor had comparable levels of precision at 46% and 47% respectively. However, BioTransformer was able to predict 90% of all experimentally confirmed metabolites, which is significantly higher than the 61% predicted by ADMET Predictor. Figure 7 illustrates some examples of CYP450-generated metabolites predicted only by BioTransformer, and others predicted only by ADMET Predictor. Details of the evaluation are available in the Additional file 6.

**Table 4:** Comparative assessment of BioTransformer and ADMET predictor (Simulations Plus) in predicting single-step human CYP450 metabolism for 60 drugs, pesticides, phytochemicals, and other xenobiotics, as well as endobiotics (e.g. lipids)

	BioTransformer	ADMET predictor
True positives	162	110
False positives	188	122
False negatives	18	70
Total no. of predictions	350	232
Precision	0.46	0.47
Recall	0.90	0.61
No. of reported metabolites	180

### Comparative evaluation of BioTransformer and the EAWAG BBD/PPS system in the prediction of environmental microbial metabolism

Meteor Nexus and ADMET Predictor are not capable of predicting environmental microbial metabolism/degradation. Therefore in order to assess BioTransformer’s abilities to predict environmental microbial metabolism, we compared it to the EAWAG-BBD/PPS system using three test compounds, namely Ampicillin (an antibiotic), Nitroglycerin (a plasticizer, a drug), and Disulfoton (an insecticide), all of which (along with their metabolites) have been found in wastewater treatment plants [21, 85, 86]. The respective structures were retrieved from ContaminantDB [41]. Here, only BioTransformer’s environmental microbial transformer was used, and only a single biotransformation step was conducted for each compound. The aim of this comparison was to assess the ability of BioTransformer to reproduce the EAWAG-BBD/PPS predictions, since the rules applicable to environmental degradation were encoded using the freely accessible EAWAG Biodegradation and Biocatalysis database. Both BioTransformer and the EAWAG-BBD/PPS system were set to apply relative reasoning, and both were set to predict all microbial transformations (i.e. aerobic and anaerobic).

**Fig. 8:**
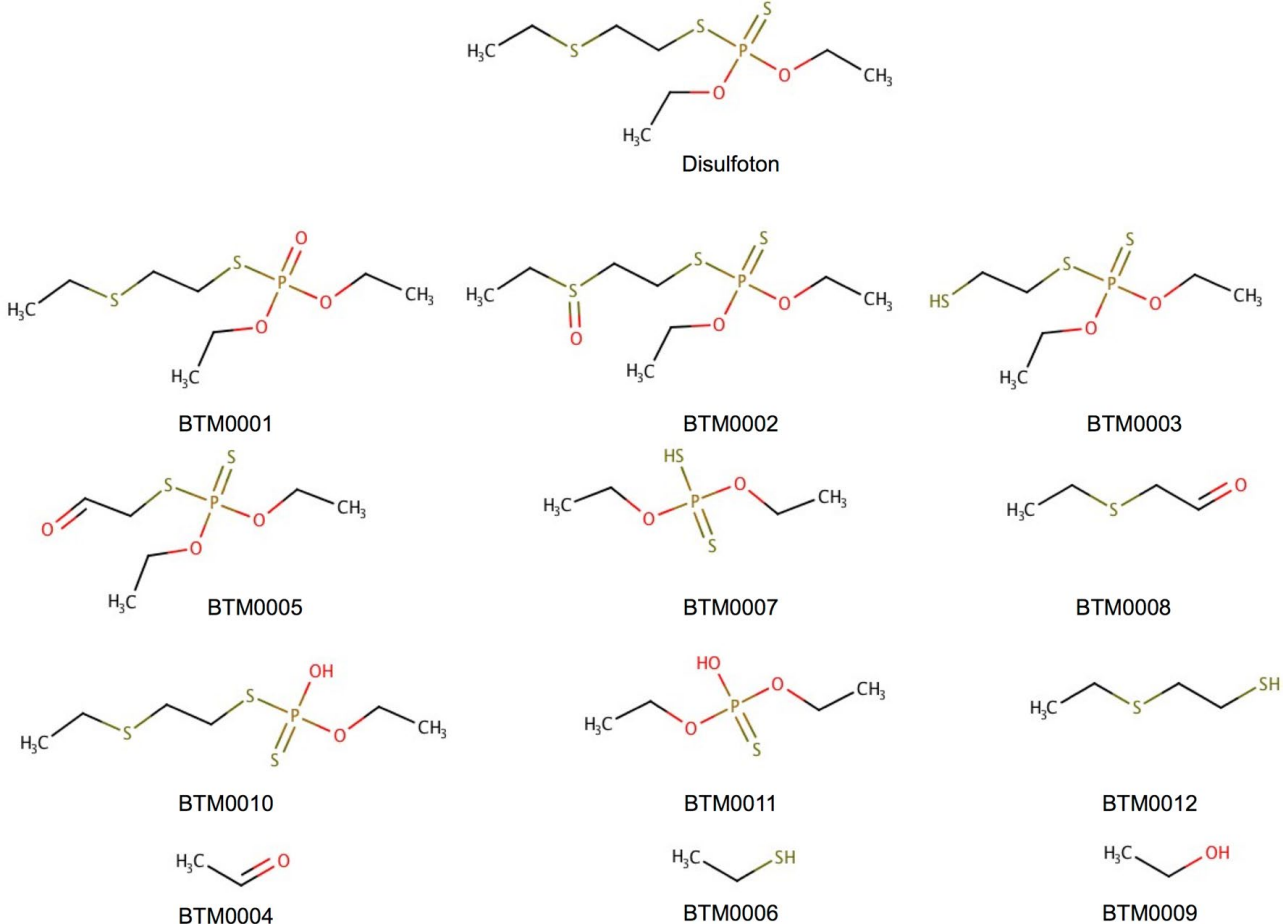
Environmental microbial metabolism of disulfoton, as predicted by BioTransformer and the EAWAG-BBD/PPS system. The metabolites BTM0004, BTM0006, and BTM0009 are reported by BioTransformer as by-products of the biotransformation bt0259 that generate BTM0003, BTM0005, and BTM0010. These by-products, which should be generated according to the rule bt0259 provided by the EAWAG-BBD/PPS, were not reported by the system

BioTransformer was able to replicate all 15 biotransformations predicted by the EAWAG system, and to successfully predict all 18 metabolites predicted by EAWAG. In addition, BioTransformer predicted three more metabolites for the degradation of Disulfoton. All three metabolites resulted from the correctly used biotransformation rule (bt0259), which was applied at three different sites of metabolism, producing two metabolites in each case. Figure 8 displays the metabolites predicted by BioTransformer and the EAWAG system, and highlights the metabolites reported only by BioTransformer.

### Evaluation of BioTransformer’s metabolite identification tool

The final evaluation of BioTransformer consisted of simply identifying putative human/mammalian metabolites of epicatechin using the BioTransformer Metabolite Identification Tool (BMIT). This was designed to simulate a real case involving the MS-based experimental analysis of epicatechin metabolites produced by rats upon a five-day treatment with epicatechin, as done by two of the co-authors of this manuscript (CM and JF). Epicatechin is an important compound from the chemical class of flavan-3-ols, and is known to exhibit cardiovascular health benefits [85–87]. It is a major component from cocoa extracts, and is also abundant in apples, grapes, berries, and tea. Briefly, rats were fed for 5 days a standardized diet supplemented with epicatechin. Spot urines were sampled after the supplementation period and compared to the spot urines sampled under the same conditions after 9 days of the same diet without epicatechin. The samples were analysed by high-resolution mass spectrometry—UPLC-QToF (Bruker, Impact II), with the mass spectrometer operated in the positive ion mode. More detailed information about the specific experimental protocols, the treatment protocols and the mass spectral data extraction/analysis is provided in Additional file 2.

**Fig. 9:**
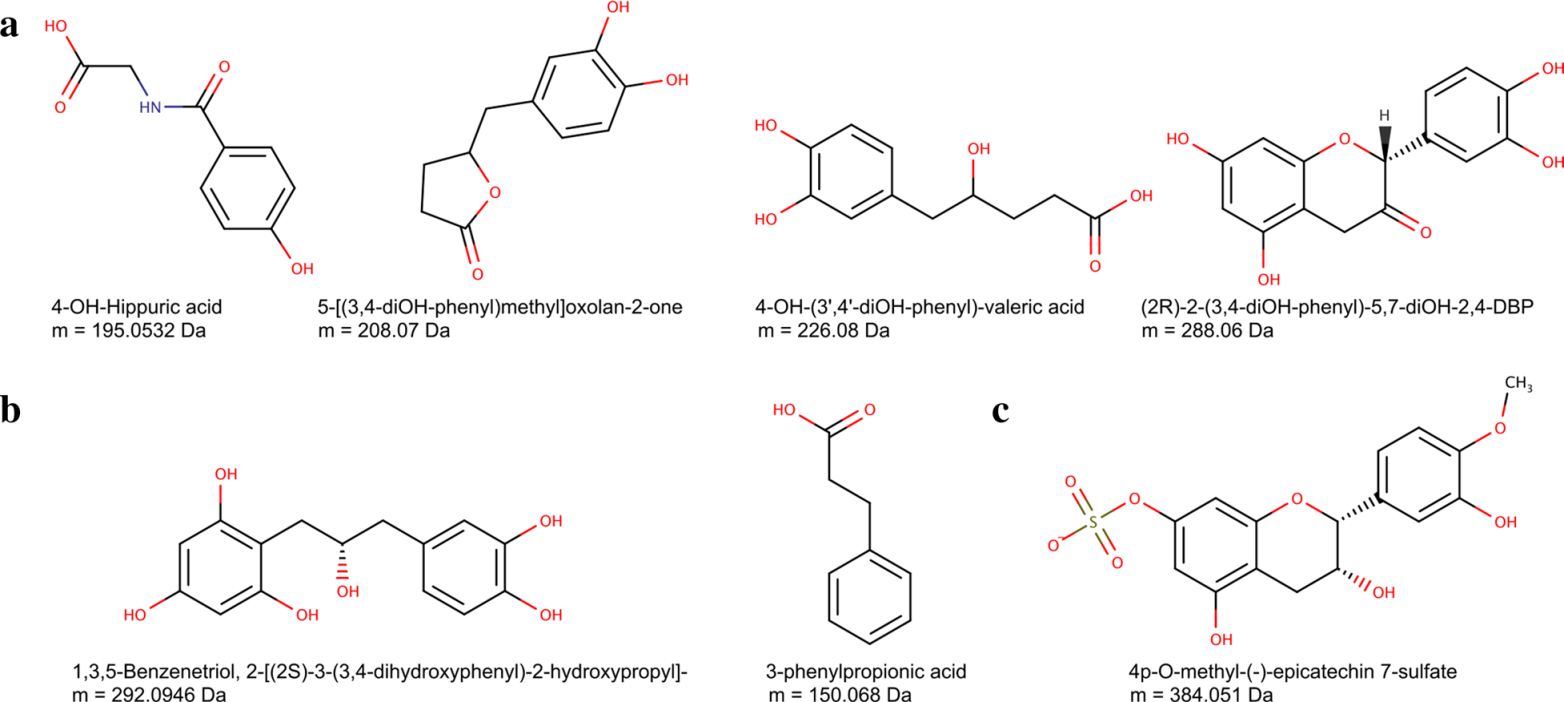
Identification of predicted metabolites of Epicatechin in humans (which are assumed to be nearly identical for rats). The figure illustrates: **a** metabolites correctly identified by BMIT, and corresponding to masses (in Da) observed in our experimental study; **b** metabolites correctly identified by BMIT, and corresponding to masses observed exclusively in previous studies, and; **c** a previously reported metabolite of epicatechin not identified by BMIT. (2R)-2-(3,4-diOH-phenyl)-5,7-diOH-2,4-DBP stands for (2R)-2-(3,4-dihydroxyphenyl)-5,7-dihydroxy-2,4-dihydro-1-benzopyran-3-one

In order to identify the metabolites observed in our study, the BMIT module used a set of 260 neutral monoisotopic masses, derived from the [M + H] + ions extracted from the experimental QToF MS data collected from the rat urine samples, ranging from 53.4896 to 969.8669 Da. Monoisotopic masses were generated by subtracting 1.00727 Da from the ions extracted from the MS dataset. Details regarding the data extraction process are provided in Additional file 2. These masses exhibited marked increases in intensity after epicatechin supplementation compared to baseline. The human supertransformer (option *superbio*) was used to facilitate putative compound identification. From the 260 monoisotopic masses that were extracted, BMIT identified 37 possible metabolites of epicatechin corresponding to 20 unique masses. These masses do not correspond to adducts or isomers and may therefore be considered parent ions (Additional file 7: Table S1). These putative identifications will have to be further investigated with MS/MS experiments and validated against authentic standards for more definitive identification. In order to acquire additional support for the identity of the predicted metabolites, the scientific literature was searched manually to collect structural data regarding epicatechin metabolites reported in previous experimental studies of both humans and rats. A total of 56 single- and multi-step metabolites of epicatechin, corresponding to 37 monoisotopic masses were identified **(**Additional file 7: Tables S1 and S2**)**. Of the 37 predicted metabolites matching our experimental data, 22 matched 11 unique and previously reported monoisotopic masses. Among those, 18 compounds corresponded to previously reported metabolites. For the nine other experimental masses that had matches with BMIT predictions, 15 possible metabolites (never previously reported) were obtained. Figure 9 shows examples of the suggested epicatechin metabolites with their masses, as identified in our study. A complete list of predicted epicatechin metabolites, along with their corresponding metabolic pathways leading to each metabolite are available in Additional file 8. Moreover, metadata (e.g. masses, retention times), and comparisons to previously reported data, can be found in Additional file 7: Table S1.

We also tested whether BMIT could identify any of the remaining 38 known metabolites (corresponding to 26 unique masses) previously reported, but not observed in our study, or not selected by our data treatment parameters. The 26 unique masses were provided to BioTransformer as input, and the identification was performed using the same mass tolerance as before (0.01). BMIT was able to suggest 28 molecules for 19 unique masses. Among those, 21 compounds corresponding to 18 unique monoisotopic masses had previously been reported as epicatechin metabolites (Additional file 7: Table S2). Figure 9 illustrates a number of epicatechin metabolites exclusively reported in previous studies, which were correctly identified by BMIT (Fig. 9b), as well as a previously reported metabolite that was not identified by BMIT (Fig. 9c). BMIT’s identification results are available in Additional file 9, and their comparison to previously reported data are available in Additional file 7: Table S2.

Overall, BMIT was able to suggest 39 epicatechin metabolites that were previously reported in the literature, 18 of which were observed in our study. Moreover, BMIT suggested 28 epicatechin metabolites that had not been reported in previous studies (17 corresponding to masses that do not match previously reported ones, and 11 extra structures matching previously known masses).

## Discussion

### BioTransformer’s design and implementation

BioTransformer is a software tool that combines both a knowledge-based approach and a machine learning approach to predict the metabolism of small molecules, and to assist in metabolite identification. The knowledge-based system consists of a biotransformation database (MetXBioDB), a knowledgebase (the reaction knowledgebase), and a reasoning engine. MetXBioDB is a unique resource that is freely available, and covers a wide range of enzymatic reactions that take place in human tissues, the human gut and the environment (soil and water microflora). In contrast to most publicly available databases, MetXBioDB provides detailed biological and chemical information about all of its biotransformations, including the catalyzing enzymes, the substrates, the products, and the biotransformation rule(s) that is/are applied. MetXBioDB describes the metabolism of > 2000 compounds catalyzed by ~ 15 enzyme families. For each biotransformation, at least one scientific source or reference is provided. MetXBioDB is stored as a JSON document, which can be easily parsed.

One potential application of MetXBioDB is in the design of biotransformation rules with narrow specificity, which can be used for in silico metabolism prediction. In fact, this resource has already been used (in addition to other data) to successfully design > 300 biotransformation rules, which were used to annotate the biotransformations in the database and predict metabolites via the BioTransformer Reasoning Engine. Despite the aforementioned strengths of MetXBioDB, the database still has a number of limitations. Although it covers a large number of enzymatic reactions, it is clear that more data is needed in order to cover an even larger set of reactions (e.g. oxidation reactions) catalyzed by enzymes other than CYP450s. It is also clear that there is a need to define more constraints and/or build additional models that would increase the quality of the predictions. Moreover, users could benefit from data about the different sites of metabolism for each specific biotransformation, as it would serve as a training set for the development of models for the prediction of sites of metabolism (SoMs). For the current version of MetXBioDB, the intent was simply to provide an easily readable and comprehensible data set. However, providing MetXBioDB in a database format that can be parsed and queried in a more sophisticated way (e.g. SQL) would make the database much more useful to a broader number of users. Efforts are underway to do so for the next release of MetXBioDB. We welcome and encourage contributions in regard to the curation, improvement, and expansion of this resource.

#### Evaluation of BioTransformer’s predictions

In our first test, BioTransformer was evaluated against Meteor Nexus (v.3.0.1). Meteor Nexus is a commercially available software tool that is considered to be the gold standard for predicting biotransformations of xenobiotics. While BioTransformer achieved a better prediction (49%) and recall (88%) than Meteor Nexus at the equivocal level of confidence (35% precision, and 71% recall), Meteor Nexus’ precision improved significantly at the plausible (56%), and probable (59%) levels. The increase in Meteor Nexus’ precision matched our expectations, as the minimum likelihood threshold for metabolite selection increased, thus reducing its probability of selecting unconfirmed metabolites. However, the 68% increase in precision (from Equivocal to Probable) led to an 82% decrease in recall. As a consequence, while Meteor Nexus’ predicted a higher percentage of true metabolites at these levels, compared to BioTransformer, it returned a significantly lower number of true metabolites.

It is worth noting that BioTransformer heavily relies on the selective nature of the biotransformation rules and other structural constraints, in addition to its implementation of relative reasoning. On the other hand, Meteor Nexus combines the continuous absolute scoring of biotransformations with relative reasoning, providing binned data for different levels of reasoning through a more dynamic scoring system. Overall, the performance of BioTransformer suggests that the freely accessible BioTransformer tool could be used to assist scientists in various drug discovery and environmental safety studies.

In our second test, we evaluated BioTransformer’s performance in predicting single-step human and human gut microbial metabolism of 20 endobiotics and xenobiotics. Overall, 69% of BioTransformer predictions matched experimentally confirmed metabolites. Moreover, BioTransformer was able to predict 87% of all reported (and experimentally confirmed) metabolites. The better performance, compared to the first test, can be partly explained by the fact that some endobiotics, such as sphingo- and glycerophospholipids, follow very classical and well-known metabolic pathways (Additional file 2: Fig. S3), which were encoded in the reaction knowledgebase. However, these compounds represent only 15% of the second test set. Therefore, these results still show that BioTransformer was also able to accurately predict the metabolism of compounds with a more complex metabolism (Fig. 7). In fact, BioTransformer was able to correctly predict the human and human gut metabolism of polyphenols (e.g. Epicatechin), and pharmaceuticals (e.g. L-DOPA). This is very promising, as little is known about gut microbial metabolism of those classes of compounds. Even for the well-studied, and biologically relevant class of polyphenols, a lot of experimental work is needed to validate the metabolic pathways for hundreds of known compounds. BioTransformer could be used to provide accurate suggestions about the identity of their metabolites and propose metabolic pathways, which could then in turn be validated experimentally.

The third evaluation involved the comparative assessment of BioTransformer’s and ADMET Predictor’s capabilities to accurately predict CYP450 metabolism of 60 pharmaceuticals, pesticides, food metabolites, and other endogenous and exogenous compounds. The comparable precision of BioTransformer and ADMET Predictor (46% and 47%, respectively) shows that on average, about half of their predictions matched experimentally confirmed metabolites. However, BioTransformer was able to predict 90% of all experimentally confirmed metabolites, which is significantly higher than the 61% predicted by ADMET Predictor.

Overall, the first three tests demonstrate BioTransformer ability to accurately predict human and human gut microbial metabolism for a very diverse set of metabolites, covering endogenous metabolites, pharmaceuticals and personal care products, food compounds, as well as other exogenous compounds. The comparative assessments of BioTransformer with Meteor Nexus and ADMET Predictor show that while BioTransformer is slightly slower, it consistently performs better, and it also addresses some of their shortcomings. In particular, BioTransformer is open access, and it covers a much wider range of chemical substrates and metabolic biotransformations.

In order to evaluate BioTransformer’s ability to predict environmental metabolism, we compared its prediction results with the EAWAG-BBD/PPS system. It is worth noting that the biotransformation and preference rules we encoded in BioTransformer were based on the same set of rules defined by the EAWAG-BBD/PPS. The key difference was that the rules were encoded in the same common SMIRKS/SMARTS format used by all of BioTransformer’s other transformer tools. Based on the sample tests provided in the Results section, it is clear that BioTransformer was able to accurately replicate the predictions provided by the EAWAG-BBD/PPS system. These results suggest that BioTransformer could also be used to accurately predict environmental microbial metabolism.

In a fourth test, we evaluated BioTransformer’s ability to identify metabolites using its BMIT module. This task tacitly relies on the metabolism prediction task, and BioTransformer was able to suggest 37 metabolites matching 20 masses from a list of 260 monoisotopic masses extracted from the MS analysis of urine samples collected after exposure to epicatechin (Additional file 7: Table S1). Of those, 18 metabolites were identified as previously known metabolites. Twenty-six monoisotopic masses matching to 36 reported epicatechin metabolites were not observed in our experimental study. This variation in the observed metabolites may be caused by different experimental settings and analytical conditions (e.g. length of the treatment, species, gender, dietary background, sample preparation and analysis methods) in different studies. For example, rats are expected to perform less sulfonation of epicatechin than humans [87]. In a second run, BMIT was used to search metabolites corresponding to monoisotopic masses that were observed in previous studies but not in our experimental dataset. In this test it was able to correctly identify another 21 known epicatechin metabolites. Overall, BMIT was able to predict 39 out of 56 previously reported compounds. The discrepancy between the number of metabolites suggested by BMIT and the number of previously reported metabolites could be explained by several factors. First, ten of the known epicatechin metabolites not predicted by BMIT (3 masses observed in our study) are products of a 2-step conjugation, but the *superbio* option simulates only one conjugation step, as it is often sufficient to make a molecule stable and hydrophilic enough for excretion (based on experimental data from MetXBioDB).

Second, in some cases (e.g. mass = 195.0532 Da), BMIT predicted two isobaric metabolites, but only one peak (retention time = 5.94 min.) was found in the spectra, indicating that only one metabolite was present in the sample or that the analytical conditions did not allow the resolution of isobaric compounds (Supplemental Table 1). Often, the same reaction (especially conjugations) can occur at several locations within a molecule, thus producing regioisomers. The opposite was seen in the case of mass = 314.064 Da, which corresponds to 3 predicted metabolites (glucuronic acid conjugates), with 5 observed peaks exclusively found in samples collected after exposure to epicatechin at 8, 11, 11.40, 11.64, 11.75 min. These examples illustrate a common problem with metabolism prediction in the identification of the correct sites of metabolism. We believe that increasing the number of true positives, as well as reducing the number of false positives could be achieved by integrating models that more accurately predict sites of metabolism.

BMIT was able to identify metabolites such as (2R)-2-(3,4-diOH-phenyl)-5,7-diOH-2,4-DBP (Fig. 9a), and other conjugated metabolites corresponding to masses not previously reported. It is worth mentioning that these are only putative predicted metabolites, and that the results of the BMIT must be validated experimentally, through further MS-based investigations. However, it was beyond the scope of this particular experimental study to fully investigate the metabolism of epicatechin. Indeed, we believe that complementary analytical platforms such as GC–MS would be necessary to cover the whole chemical space of epicatechin metabolites. Thorough identification of the observed metabolites using MS/MS or authentic (synthesized) standards was not performed in our assessment of the metabolites present in urine. Epicatechin is metabolized in the liver, and more extensively by the gut microbiome. The ability of BMIT to identify/predict both human and human microbial epicatechin metabolites suggests that this module would be a useful asset in elucidating the dark matter in host-microbiome metabolomics [88]. BMIT should also be a very useful tool for general metabolism prediction and metabolite identification using MS or MS/MS data. In addition, the predictions generated by BMPT could be very useful for suspect-screening analysis, and thereby permit faster non-targeted data analysis and more facile putative compound identification. Thanks to in silico MS/MS fragmentation tools such as CFM-ID, the computation of MS/MS-spectra for those metabolites could be used to provide additional evidence.

We believe the examples used here nicely demonstrate the ability of BioTransformer to accurately predict a wide range of metabolic reactions, for a number of different types of small molecules (endogenous and xenobiotic compounds) and a number of different biosystems (humans, microbial/environmental). BioTransformer is unique in its ability to cover almost all aspects of non-essential metabolism (drug/xenobiotic metabolism, endogenous compound metabolism, gut microbial metabolism, environmental metabolism). This makes it particularly useful for the wide-ranging applications seen in metabolomics and other small molecule studies. Furthermore, the accuracy, coverage, precision and recall of BioTransformer appear to be as good as, or even much better than some of the most highly regarded metabolic prediction systems now available. It is also notable that BioTransformer, unlike most of its competitors, is freely available.

Certainly a more extensive analysis of a much larger set of query compounds would likely better illustrate the strengths and weaknesses of BioTransformer. However, it is important to remember that there are relatively few experimentally validated, comprehensive sets of metabolic “biotransformation trees” and that the examples selected here (which required hundreds of hours to assemble, curate and validate) cover a good portion of the better known trees.

While there are a number of strengths to BioTransformer, we believe that certain improvements could still be made to the program. First, the addition of more biotransformation data would certainly provide additional reaction “fodder” to create more biotransformation rules. Additional biotransformation data would also provide further statistical evidence to fine tune the reaction preference rules (relative reasoning) and occurrence ratios for absolute/relative reasoning. In particular, adding an option for absolute reasoning would give BioTransformer the ability to select candidates with a set cut-off score. Currently BioTransformer’s biotransformation database (MetXBioDB) and its reaction knowledgebase cover only a small portion of gut microbial metabolism (i.e. metabolism of plant-derived polyphenols). As many xenobiotics as well as endogenous compounds are known to be metabolized in the gut [75, 89–92], it will be important to further expand the coverage of gut microbial metabolism in BioTransformer. We plan to make these improvements in upcoming versions of BioTransformer. Over the longer term we are hoping to integrate more machine learning prediction models (e.g. SoMs for CYP450 metabolism, and SoMs for phase II metabolism). This integration depends mostly on the amount of data available as machine learning hinges on having large and diverse training sets to optimize its performance. Given that the number of experimentally confirmed biotransformations is still quite low for the systems of interest, it is likely that this will take a number of years to complete.

## Conclusion

In this work, we have presented BioTransformer, a freely available, open access software tool that supports the rapid, accurate, comprehensive prediction of metabolism of small molecules in both mammals and environmental microorganisms. BioTransformer can also assist in metabolite identification using experimental MS data. BioTransformer can be used either as a command-line tool or as an imported library. The Java executable and Java library are open access, and freely available at https://bitbucket.org/djoumbou/biotransformerjar/. Moreover, BioTransformer is also freely accessible as a web service at www.biotransformer.ca. The web service provides users with the possibility to manually or programmatically submit queries, and retrieve data generated by the BioTransformer software tool.

Within mammals, we have shown that BioTransformer was able to accurately predict single-step biotransformations for a diverse set of xenobiotics, including drugs, pesticides, and food compounds. The reactions that BioTransformer predicts cover Phase I and Phase II metabolism in mammals, as well as the human gut microbial metabolism. Overall, BioTransformer was shown to perform better than Meteor Nexus and ADMET Predictor, two highly regarded commercial software tools for in silico metabolism prediction. Unlike most other metabolic prediction tools, BioTransformer also supports the prediction of metabolism of small molecules by environmental microbes. The integration of environmental metabolism with endogenous human and gut microbial metabolism allows BioTransformer to address many of the predictive metabolic needs of metabolomics or exposomics researchers, which tend to span a much wider range than, say, drug researchers, food chemists or environmental scientists.

Despite its strengths, BioTransformer is not without some limitations. Addressing these would certainly make the program much more flexible, more accurate, and more comprehensive. Obvious improvements for the current version of BioTransformer include: (1) the validation of BioTransformer’s predictions for a larger and more diverse test set of molecules; (2) the experimental validation of BioTransformer’s BMIT predictions for a larger set of molecules and experimental data; (3) the expansion of the reaction knowledgebase to cover more reactions, and (4) the addition of new options for metabolite prediction/ranking.

## Additional files

**Additional file 1.** Cited structures.

**Additional file 2.** Additional-Notes-Introduction-Methods-Evaluation.

**Additional file 3.** Phase-II-Filter-Features.

**Additional file 4.** Predictions: BioTransformer vs. Meteor Nexus.

**Additional file 5.** BioTransformer—human and human gut microbial metabolism.

**Additional file 6.** BioTransformer vs. ADMET Predictor.

**Additional file 7.** Epicatechin metabolite identification tables.

**Additional file 8.** Epicatechin metabolites identification part 1.

**Additional file 9.** Epicatechin metabolites identification part 2.

## Abbreviations

ADMET: absorption distribution metabolism excretion toxicology
BMIT: BioTransformer metabolite identification tool
BMPT: BioTransformer metabolism prediction tool
CYP450: cytochrome P450
CSV: comma-separated values
EC: enzyme classification
JSON: JavaScript object notation
KB: knowledgebase
PPC: pharmaceutical and personal care product
SDF: structure data file
SMILES: simplified molecular-input line-entry system
InChI: international chemical identifier
SULT: sulfotransferase
UGT: UDP-glucuronosyltransferase
