# Supplementary material for: BioTransformer: a comprehensive computational tool for small molecule metabolism prediction and metabolite identification
Source: J Cheminform. 2019 Jan 5;11:2. doi: 10.1186/s13321-018-0324-5 (PMC6689873; doi:10.1186/s13321-018-0324-5)
Supplement: Supplementary file 2 — Additional file 2. Additional-Notes-Introduction-Methods-Evaluation. [file 13321_2018_324_MOESM2_ESM.docx]

**Additional File 2**

BioTransformer: A Comprehensive Computational Tool for Small Molecule Metabolism Prediction and Metabolite Identification

Yannick Djoumbou-Feunang^1^, Jarlei Fiamoncini^2,3^, Alberto Gil de la Fuente^4^, Russell Greiner^5,6^, Claudine Manach^2^, David S. Wishart^1,5^

^1^Department of Biological Sciences, University of Alberta, Edmonton, Alberta, Canada, T6G 2E9

^2^INRA, Human Nutrition Unit, Université Clermont Auvergne, F63000 Clermont-Ferrand, France

^3^Department of Food and Experimental Nutrition, School of Pharmaceutical Sciences, University of São Paulo, São Paulo, Brazil

^4^Department of Information Technology, CEU San Pablo University, Madrid Spain

^5^Department of Computing Science, University of Alberta, Edmonton, Alberta, Canada, T6G 2E8

^6^Alberta Machine Intelligence Institute, University of Alberta, Edmonton, Alberta, Canada T6G 2E8

Corresponding author: David S. Wishart, Department of Biological Sciences, University of Alberta, Edmonton, Alberta, Canada, T6G 2E8, Tel: (780) 492-0383, Fax: (780) 492-

**Methods**


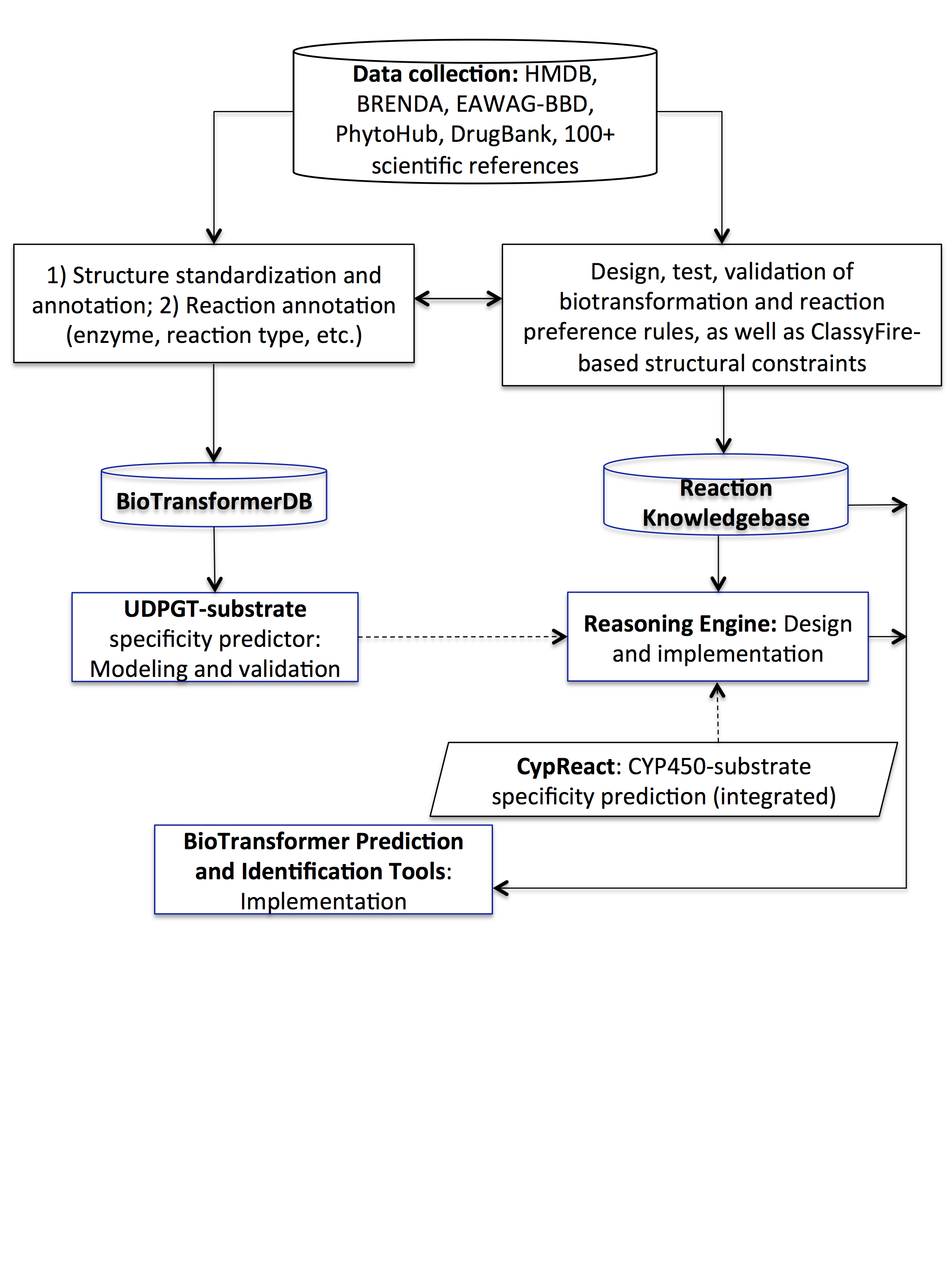


Fig S1 An illustration of the general workflow, design and implementation of the main components within BioTransformer.

*MetXBioDB: Data collection and curation*

The data curation process was conducted collaboratively with a small team of chemistry experts, and consisted of three phases. These phases involved: 1) the collection of biotransformation data, 2) the creation and annotation biotransformation objects and, 3) data validation.

Enzyme-substrate associations were collected from various publicly available databases including DrugBank (1), SuperCYP (2), PharmGKB (3), XMETDB (4), PhytoHub (5), and Phenol-Explorer (6). Other enzyme-substrate associations were extracted manually from >100 scientific papers, review articles and drug metabolism textbooks, most of which were accessible electronically via the PubMed Central (7). When available, information about the structure and/or the name of the metabolite was also extracted. In some cases, insufficient information was provided about the exact reaction type, the structure of the resulting products, or the site of metabolism. Moreover, for several compounds the reported sets of metabolites were either incomplete or conflicting between reports. Such scenarios required further reading and checking by the annotation team in order to acquire more supporting evidence, and to further validate the data. During the data collection and validation process, enzyme associations were retained only if they had experimental supporting evidence about the correct structure of the metabolite, or both the site of metabolism and the reaction type. In total, 782 enzyme-substrate associations were validated. For each biotransformation, the reactant and products were required to have a valid name and valid structural representations (SMILES string and standard InChIKey). The InChIKeys proved to be very useful for sorting, grouping and categorizing, as well as in the indexing and searching of the chemical database. For most compounds the structures were available from online databases such as DrugBank (8), ChEBI (9), PubChem (10), and PhytoHub (5). When necessary, structures were generated using ChemAxon’s MarvinSketch v.17.2.27.0 (11). In many cases the same compound was found to have several identifiers (e.g. names, synonyms IDs, etc.) and several structural representations that were linked to the same name, due to the existence of multiple salt forms, different protonation states, and tautomerism. This is a very common problem in managing chemical information, and represents a significant challenge in chemical data curation and aggregation. In an effort to eliminate this problem, all the structures were standardized through the removal of salts and charges. For mixtures, only the active compound was selected. Particular attention was also paid to stereochemistry, when the information was provided. After the name and structure standardization process was complete, a list of unique compounds was created by comparing the standard InChIKeys and aggregating the data corresponding to each InChIKey. If no name was reported for a given reactant, product or metabolite, additional online databases were searched using the standardized structure until a name was found. If a given name could not be found, an appropriate chemical name was generated using ChemAxon’s MarvinSketch.

In certain simple cases, the name of the metabolite could be derived from information about the site of metabolism and the type of reaction (e.g. 3-OH glucuronide for a glucuronidation of the hydroxyl group at the C3 position). For each compound, identifiers from external databases were also collected, using DataWrangler. DataWrangler is an in-house chemical annotation tool, which searches four major chemical databases for the given small molecule and returns various types of data, including links to other databases. The reaction type for each assembled reaction or biotransformation was assigned by selecting the corresponding biotransformation rule in BioTransformer’s reaction knowledgebase. When the corresponding reaction type or pattern was unavailable, a new metabolic reaction object was added to the knowledgebase. Certain enzyme classes, such as CYP450s, have very broad substrate specificity, and can catalyse a large pool of reactions. Therefore, it is common that a phase I oxidative transformation of a small molecule will be mediated by several CYP450s, one of which would be the major catalysing enzyme. Additionally, it is often the case that several reactions would apply to a single starting compound, leading to different metabolites (and metabolic pathways), at least one of which would be the major pathway. When available, such information was also integrated into the database. The validation process consisted of having one or more database curators check the correctness of the structures, names, reaction types and enzyme lists. For each reported biotransformation, a list of scientific sources providing supporting evidence was compiled. Because one of the main goals of MetXBioDB is to support the development of *in silico* metabolism prediction models, the biotransformations had to be accurately reproduced when applying the specified reactions. Considerable effort was put into performing this specific task, as described in the section about the reaction knowledgebase, which also led to improvements in the encoded reaction descriptions in the reaction knowledgebase. All the data in MetXBioDB is stored as a JSON document. It currently contains 1,716 unique enzyme-reactant associations, 2,199 unique biotransformations, and 1,258 unique external database identifiers for a total of 3,437 unique compounds.


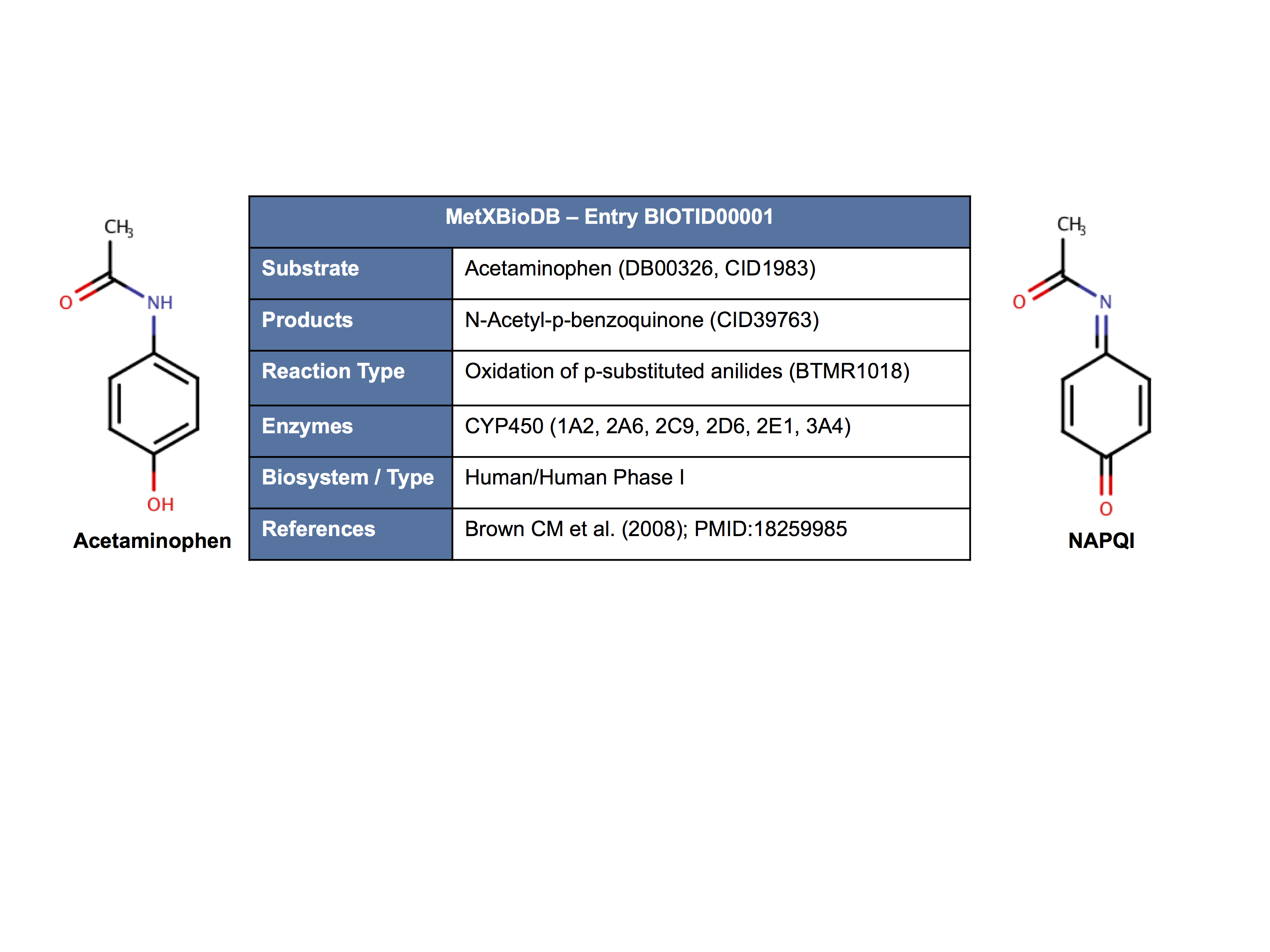


Fig S2 Example of an entry (BIOTID00001) in MetXBioDB. The database covers human CYP450/phase I, phase II, and human gut microbial metabolism. It contains over 1200 biotransformations, 2,800 enzyme-reactant associations, and 1,300 external database identifiers for more than 1,400 compounds.

*The Reaction Knowledgebase*

A typical reaction scheme encoded in the reaction knowledgebase is shown in Figure S4, which illustrates the biotransformation of 1,2-dihexanoyl-sn-glycero-3-phosphoserine (PS(6:0/6:0)) into 1,2-dihexanoyl-sn-glycero-3-phosphoethanolamine (PE(6:0/6:0)) by human phosphatidylserine synthase 2 (EC 2.7.8.29). The encoding of this generic reaction via SMIRKS and SMARTS allows the reaction to automatically replace the ethanolamine in any diacyl-sn-glycero-3-phosphoethanolamine by a serine molecule to produce the corresponding diacyl-sn-glycero-3-phosphoserine.


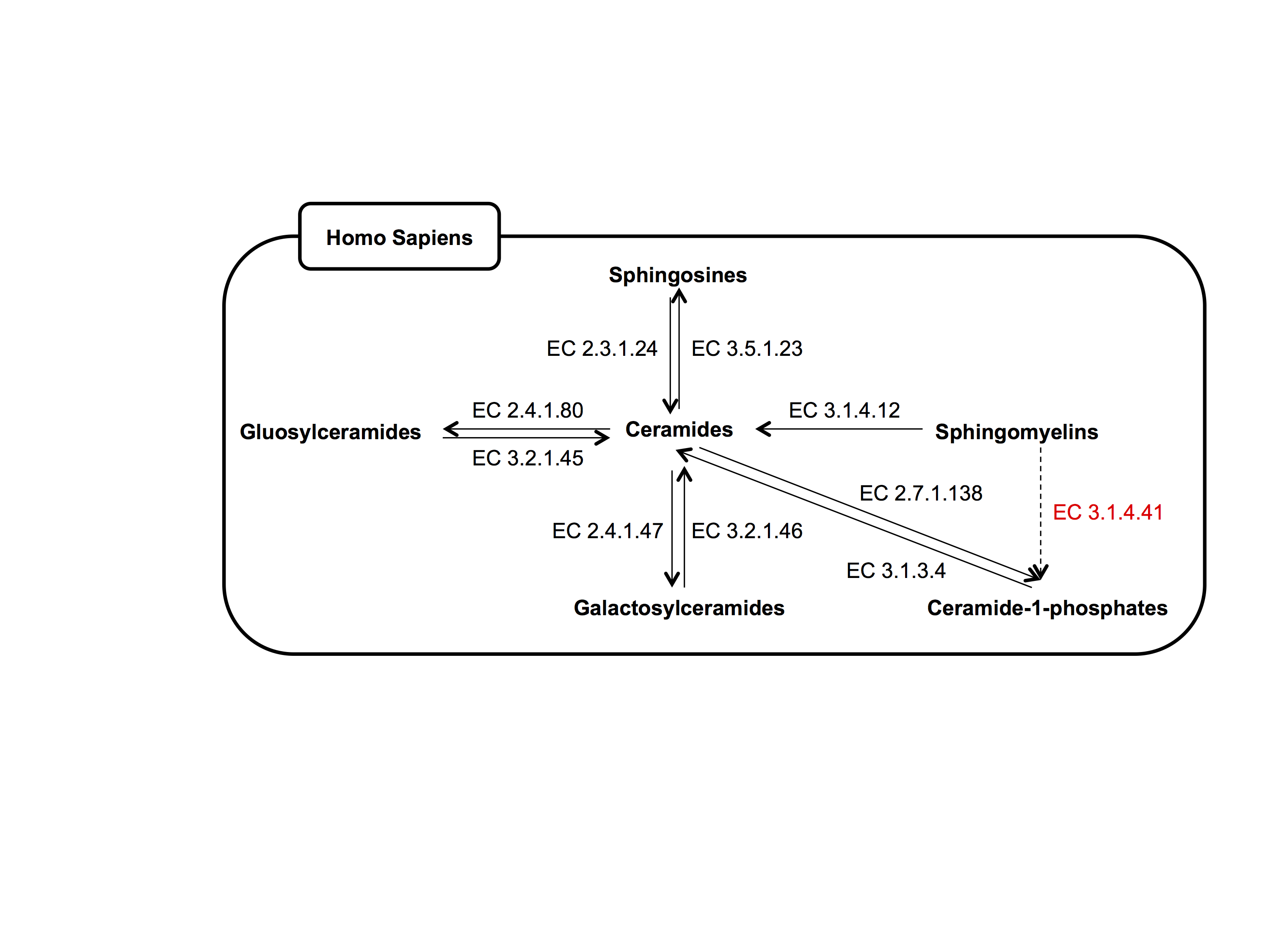


Fig S3 Interrelationships between the five different concepts represented in BioTransformer’s Reaction Knowledgebase. The figure depicts a small portion of the sphingolipid metabolism pathway in humans, as provided by the KEGG database. An example of a metabolic reaction is the conversion of compounds from the chemical class of sphingomyelins into their corresponding ceramides (as shown by the corresponding arrow) by the enzyme sphingomyelin phosphodiesterase (EC 3.1.4.12). The dotted arrow shows the conversion of sphingomyelins to ceramide-1-phosphates by sphingomyelin phosphodiesterase D (EC 3.1.4.41), which is expressed in Aspergillus flavus, but not in humans.


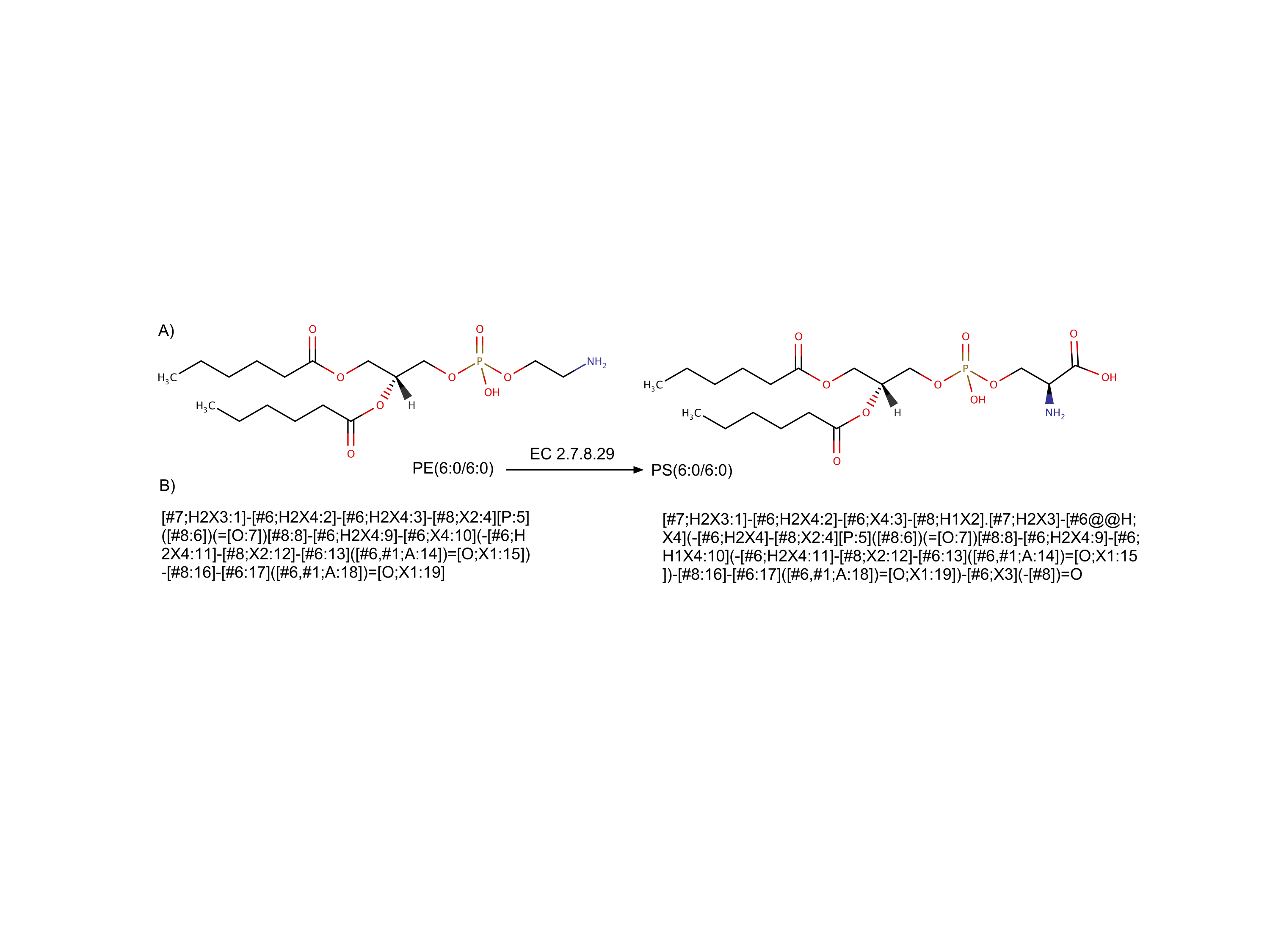


Fig S4 Encoding a phosphatidylserine biosynthetic reaction. A) Metabolism of 1,2-dihexanoyl-sn-glycero-3-phosphoethanolamine (PE(6:0/6:0)) to 1,2-dihexanoyl-sn-glycero-3-phosphoserine (PS(6:0/6:0)) by the human phosphatidylserine synthase 2 (EC 2.7.8.29). B) The encoding of the reaction in the SMIRKS language.


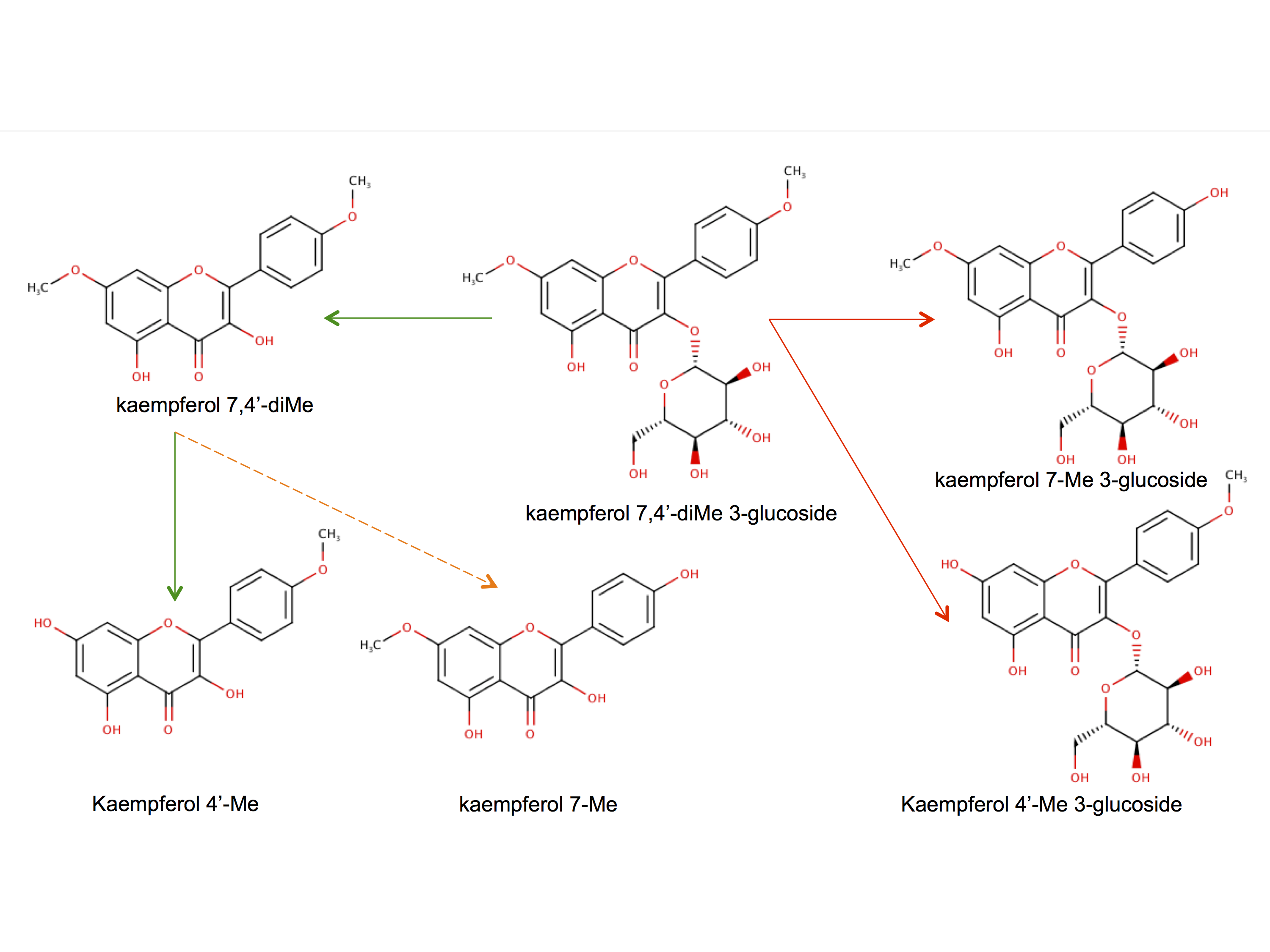


Fig S5 Metabolism of kaempferol 7,4'-dimethyl ether 3-glucoside in the human gut microbiome. The encoding of preference rules provides a more likely metabolism pathway leading to kaempferol 4’-methyl ether. Me = methyl ether; diMe = dimethyl ether.

### *BioTransformer’s Input and Workflow*


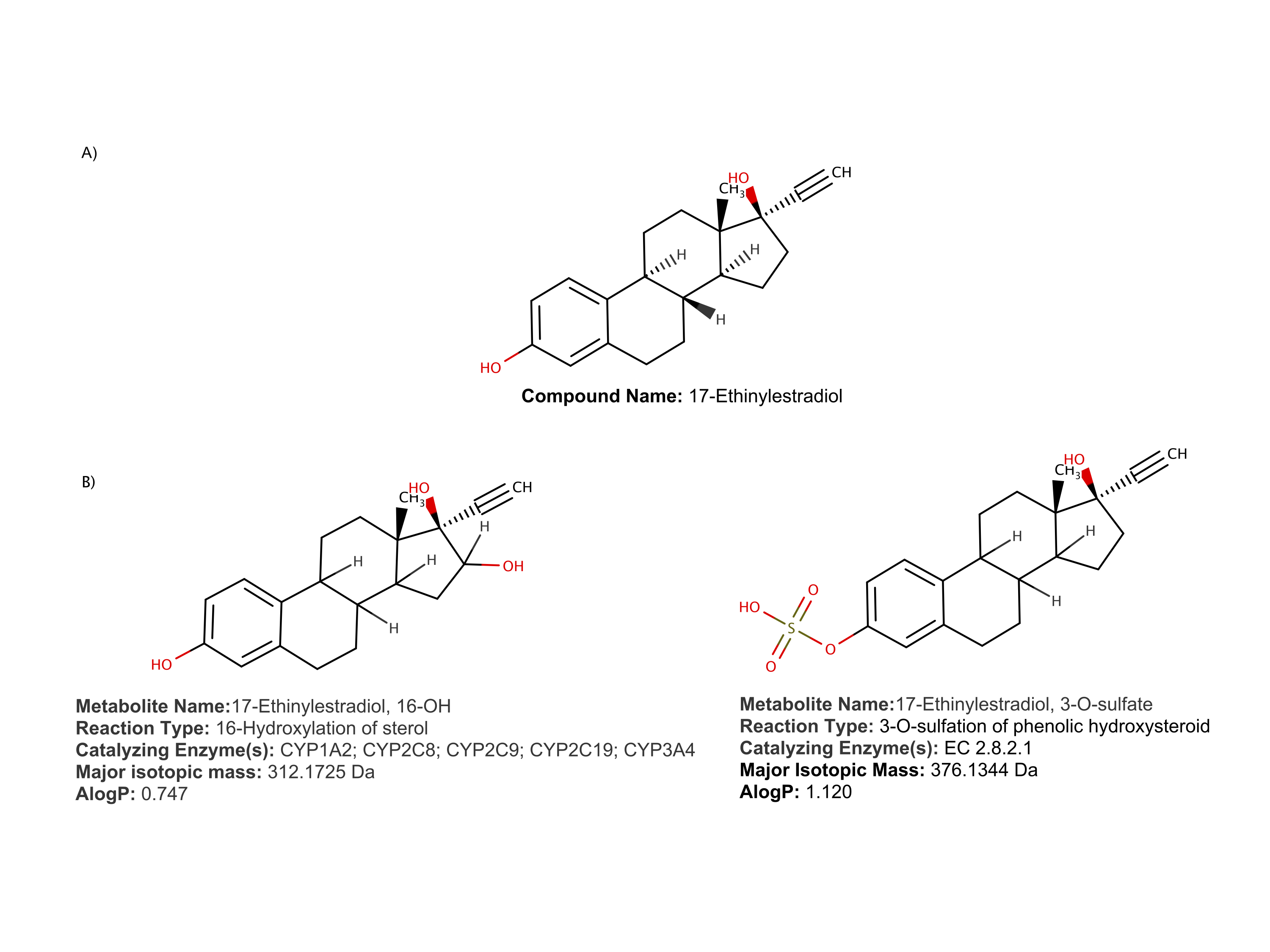


Fig S6 Metabolism prediction of 14-Ethinylestradiol. The figure illustrates examples of metabolites of 14-Ethinylestradiol (A) in the human superorganism (option *allHuman*), as predicted by the BioTransformer Metabolite Prediction Tool (BMPT). Upon prediction, BMPT provides a detailed description of each metabolite, and biotransformation (B).

### *The BioTransformer Web Server*


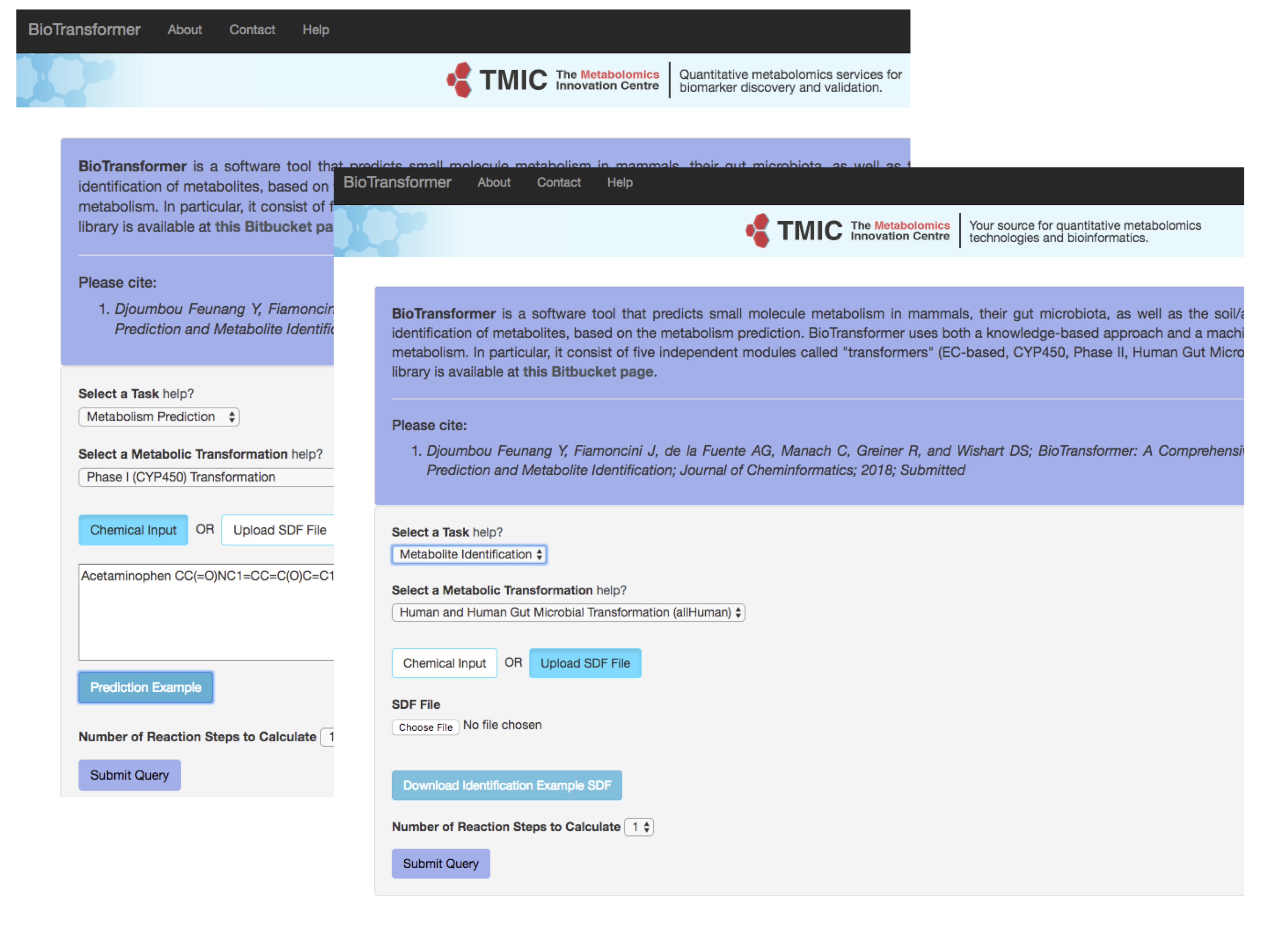


Fig. S7 BioTransformer query submission: BioTransformer accepts several input formats, including SMILES, InChI, MOL, and SD Files.


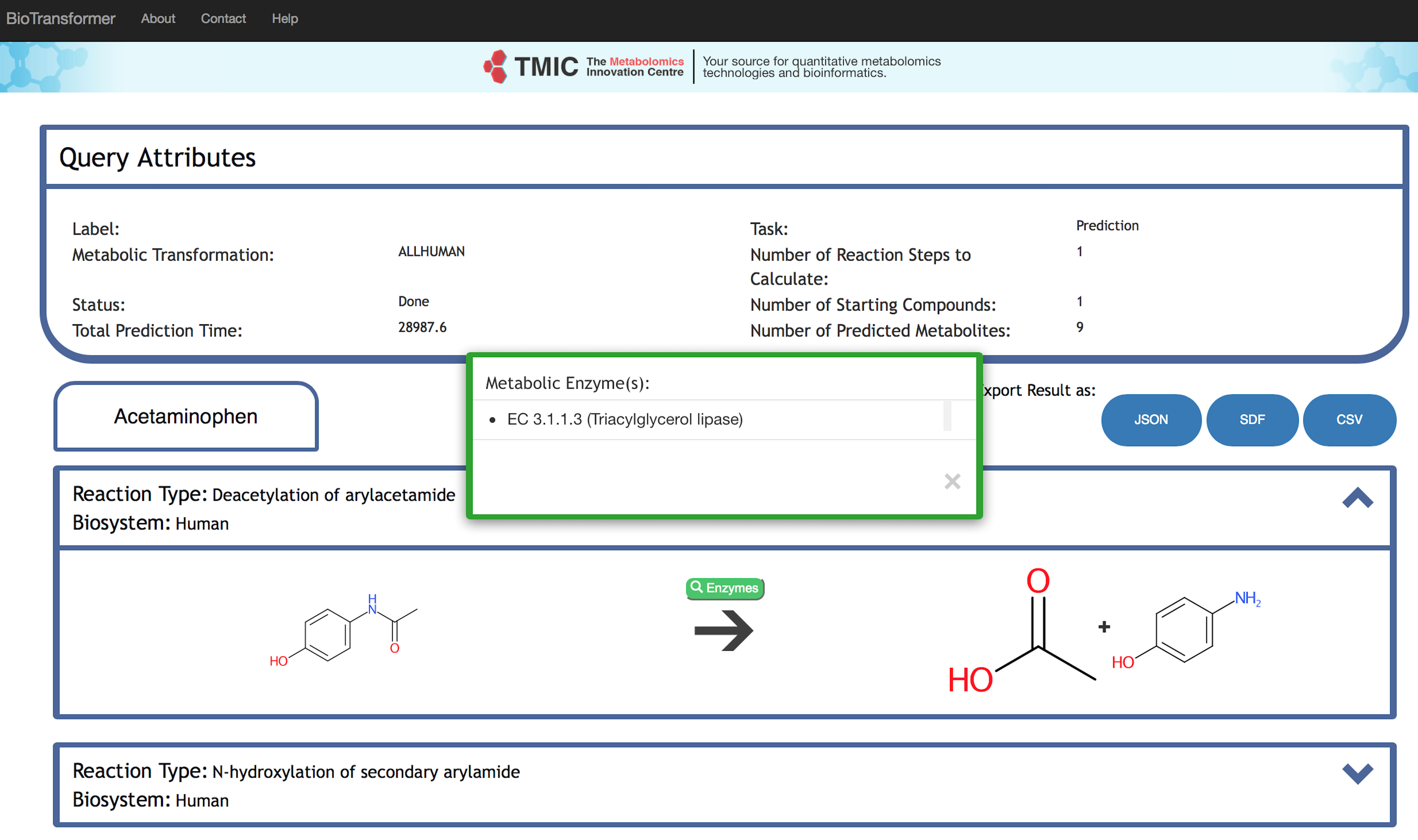


Fig. S8 Examples of metabolism prediction results. The figure illustrates the HTML document containing results for a query, upon prediction. Results are illustrated as an HTML document, and can be downloaded in JSON, SDF, or CSV format.

**Comparative Assessment of BioTransformer’s and Meteor Nexus’ Metabolism Predictions**

The first step involved a comparative assessment of BioTransformer (version 1.0.4) and Meteor Nexus (Lhasa Limited, UK), version 3.0.1 (12), in predicting the single-step human metabolism of 40 pharmaceuticals and pesticides, randomly selected from DrugBank and T3DB (8). The test set was limited to those classes since the Meteor Nexus biotransformation dictionary and associated rule bases are predicated mainly on studies of these classes of compounds. Both BioTransformer and Meteor Nexus were set to use absolute/relative reasoning to prioritize the more likely biotransformations.

**Program Setup and Settings**

The following parameters were set in BioTransformer for the prediction of single-step metabolism: Biosystems (Human); Phases (I and II); Depth (1); Prediction Method: Absolute/Relative Reasoning; Apply Relative Reasoning: YES; Max. metabolites (N/A); Min. Likelihood (N/A). The following parameters were set in Meteor Nexus for the prediction of single-step metabolism: Biosystems (Human); Phases (I and II); Depth (1); Prediction Method: Absolute/Relative Reasoning; Apply Relative Reasoning (YES; Level cutoff = 2); Max. metabolites (default = 60); Min. Likelihood (EQUIVOCAL, PLAUSIBLE, and PROBABLE); Phase constraints: Grow from Phase II. The settings of both tools are comparable and almost identical. A few test were ran on a very small number of compounds to determine optimal settings when using Meteor Nexus.

**Comparison of Metabolite Predictions**

The comparison of metabolites was performed by structure matching. It is worth noting that the stereochemistry is not always conserved. In particular, metabolite structures predicted by Meteor Nexus did not contain stereochemical information; therefore metabolites lacking stereochemistry were also accepted. In a few cases, the discrepancies in the definition/encoding of reactions were reflected in the results. Two scenarios were observed:

***Scenario 1:*** The same reaction was reported by both tools, but the set of generic products varies. Instances include the N-dealkylation of aliphatic tertiary amines (e.g. Atrazine, Donepezil, Lidocaine). In BioTransformer, the reaction results in the production of the carbonyl derivative of the aliphatic moiety and the remaining amine moiety. However, the equivalent reaction (oxidative N-dealkylation) results in the production of the alcohol, carbonyl, and carboxyl species for the aliphatic moiety, in addition to the remaining amine moiety. For instance, the N-dealkylation of Atrazine resulted in the generation of Deethylatrazine, acetone, and acetaldehyde in BioTransformer (See Figure S9). Meteor Nexus predicted acetic acid, in addition to those three metabolites, through oxidative N-dealkylation (See Figure S9). The N-dealkylation of Donezepil results in the formation of benzaldehyde and 5,6-Dimethoxy-2-(piperidin-4-ylmethyl)-2,3-dihydro-1H-inden-1-one in BioTransformer. The equivalent reaction in Meteor Nexus generated both metabolites, as well as benzyl alcohol and benzoic acid. Because the aldehyde form is the one returned by most references in the scientific literature for this type of reaction, the additional metabolites predicted by Meteor Nexus were omitted and not considered as false positives. In the case of Atrazine, Meteor did not predict the alcohol derivative of the removed aliphatic moiety, as it was the case for Donezepril, which is a bit confusing.

***Scenario 2:*** Meteor Nexus predicted a metabolite that is usually reported (in the literature) to occur upon multiple-step metabolism. This was the case for Cotinine, a metabolite of Nicotine (See Figure S10). While this metabolite is predicted by BioTransformer after two steps of metabolism (“Formation of iminium from pyrrolidine ring” followed by “Oxidation of a piperidine or pyrrolifine iminium ion intermediate”), it is predicted by Meteor Nexus upon a single biotransformation (Lactams from 2-Arylpyrrolidines) of Nicotine. The prediction of nicotine was counted as a false negative.


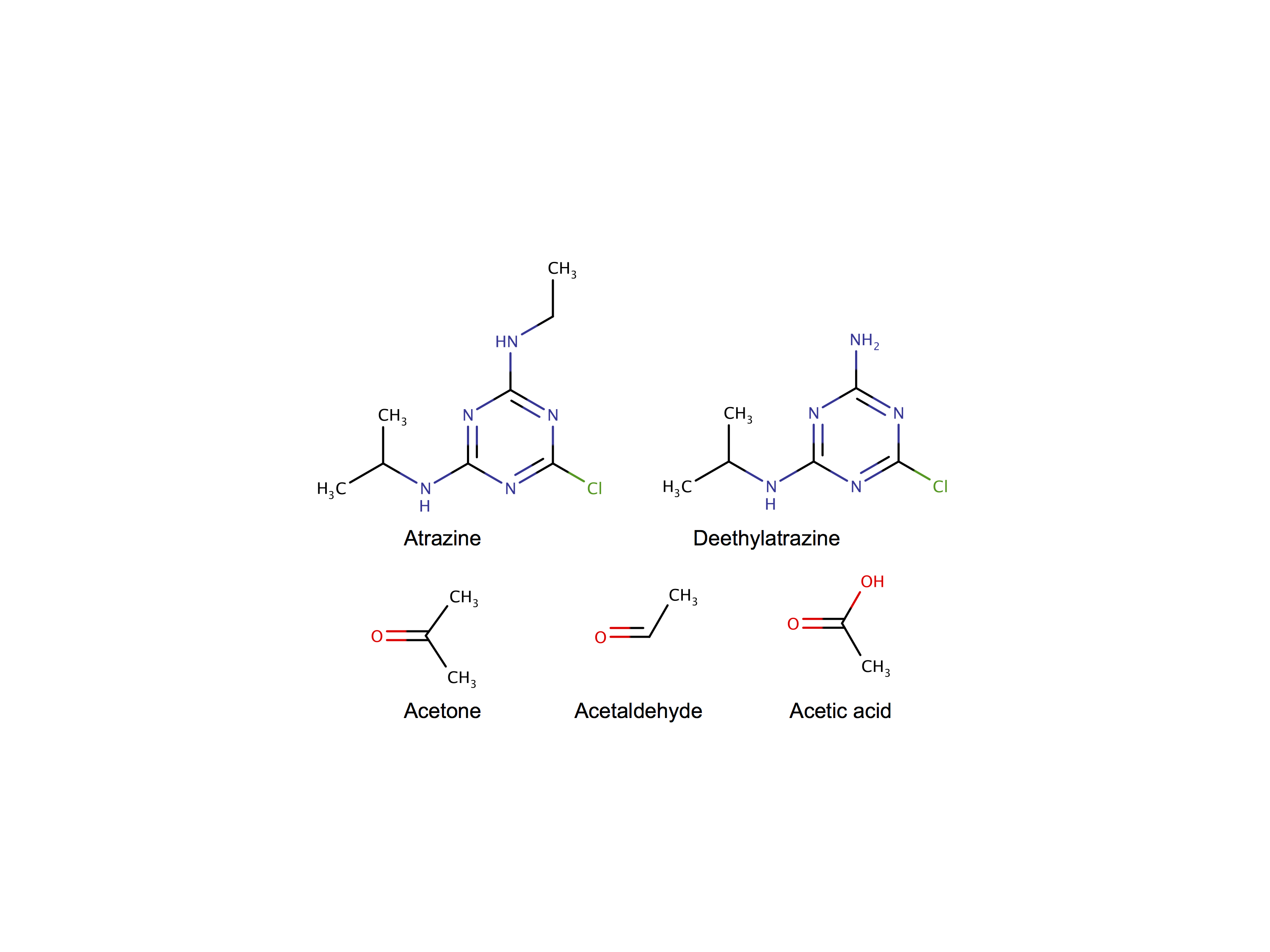


Figure S9: Discrepancies between BioTransformer and Meteor Nexus in the prediction of Atrazine metabolism. Acetic acid predicted by Meteor Nexus upon one N-delakylation (N-deethylation) of Atrazine, but not by BioTransformer.


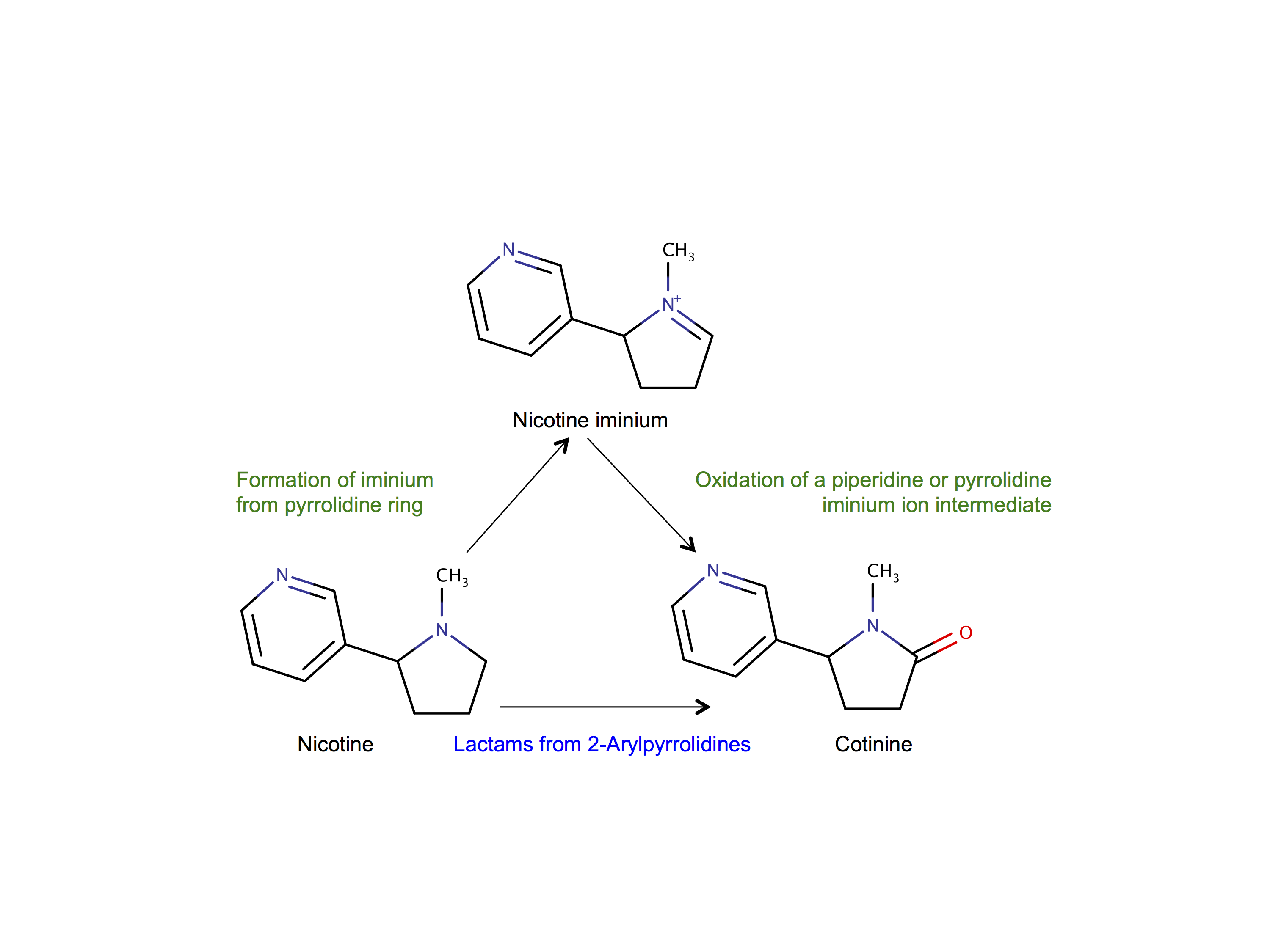


Figure S10: Discrepancies between BioTransformer and Meteor Nexus in the prediction of Nicotine metabolism.

**Experimental Mass Spectrometry Analysis of Epicatechin Metabolism in Rats**

**Animal experiments**

The animal experiments were conducted at the Unit of Human Nutrition at the French National Institute for Agricultural Research (INRA), Saint-Genès-Champanelle, France. The study design was approved by the ethics committee CEMEA Auvergne (ref – APAFIS# 10136-2015091714243190 v6 A). Briefly, 12-week old Wistar rats (2 male and 2 female) purchased at Janvier Labs (Saint Berthevin, France) were fed a chemically defined AIN-93 powder diet for 9 days (Ssniff diets, Soest, Germany). Urine was sampled by placing the rats in metabolic cages for 3 hours. During sampling, the urine container was kept in ice to avoid microbial growth and metabolite degradation. After the baseline urine sampling, the rats were fed for 5 days with the same diet added with 0.05% epicatechin (w:w). After the exposure to epicatechin, urine was sampled once more as described above. All samples were kept at -80 °C until the moment of the analysis.

**Sample analysis and data extraction**

In preparation for the mass spectrometry analysis, urine samples were 4-fold diluted with Milli-Q water and centrifuged at 12000g for 10 minutes. Equal amounts of each sample were pooled to prepare the quality control sample, used to control equipment drift. The samples were analyzed using UPHLC (Thermo Scientific, Dionex Ultimate 3000 series) coupled to a QToF MS (Bruker, Impact II) equipped with an electrospray ionization source (ESI) operating in positive mode. Five microliters of the sample were injected into an Acquity BEH Shield RP 18 100 x 2.1mm x 1.7µm column at 20 °C. Mobile phase components were A: 1% formic acid and B: acetonitrile with 1% formic acid. The column was eluted at a flow rate of 400 μL/min with a gradient of 0% B increasing from 0 to 10% over 2-7 min, followed by an increase from 10 to 95% B over 7−22 min. The mobile phase was then returned to 100% A at 22.1 min for 4 min re-equilibration. The capillary voltage, nebulizer pressure, drying gas flow and gas temperature were set to 2500V, 41psi, 9L/min and 200°C, respectively. Data from the mass spectrometer was collected in a full-scan mode from 50-1000 m/z in a scan rate of 10,000 spectra per second (10,000 Hz).

Data extraction and processing was done using Workflow for Metabolomics (W4M) (13) on the platform Galaxy. Parameters for peak picking, grouping, alignment and retention time correction using XC-MS modules included in W4M included: Centroid mode for peak extraction; 5 ppm as the maximum tolerated mass deviation; a signal to noise ratio = 5; m/z difference = 0.01; width of overlapping m/z slices = 0.01; retention time correction method = peakgroups; smoothing method = loess. A data matrix containing m/z, intensities and retention time of the different ions was obtained. In order to avoid false positive/noise, an intensity filter set at 20000 was applied. Finally, we selected only those ions absent from all control samples and observed in at least 75% of the urine samples collected after epicatechin exposure. After all these filtering steps, the total number of ions was 260. We considered these ions as putative epicatechin metabolites and as they were measured in positive mode [M+H]+, they have been converted to the corresponding monoisotopic neutral masses by subtracting 1.00727, the mass of a proton.

The metabolism of epicatechin was predicted using BioTransformer, generating a list of monoisotopic masses of the predicted metabolites, whose presence in the dataset of 260 ions was manually verified. In this study we did not confirm the identity of the predicted metabolites, but only searched for the presence of epicatechin-specific signals of high intensity matching the predicted masses.

**References**

(1) Wishart DS, Feunang YD, Guo AC, Lo EJ, Marcu A, Grant JR, et al. DrugBank 5.0: A major update to the DrugBank database for 2018. Nucleic Acids Res 2018;46(D1):D1074-D1082.

(2) Preissner S, Kroll K, Dunkel M, Senger C, Goldsobel G, Kuzman D, et al. SuperCYP: A comprehensive database on Cytochrome P450 enzymes including a tool for analysis of CYP-drug interactions. Nucleic Acids Res 2009;38(SUPPL.1).

(3) Whirl-Carrillo M, McDonagh EM, Hebert JM, Gong L, Sangkuhl K, Thorn CF, et al. Pharmacogenomics knowledge for personalized medicine. Clin Pharmacol Ther 2012;92(4):414-417.

(4) Spjuth O, Rydberg P, Willighagen EL, Evelo CT, Jeliazkova N. XMetDB: An open access database for xenobiotic metabolism. J Cheminformatics 2016;8(1).

(5) PhytoHub. 2017; Available at: [http://phytohub.eu](http://phytohub.eu), 2017.

(6) Rothwell JA, Perez-Jimenez J, Neveu V, Medina-Remón A, M'Hiri N, García-Lobato P, et al. Phenol-Explorer 3.0: A major update of the Phenol-Explorer database to incorporate data on the effects of food processing on polyphenol content. Database 2013;2013.

(7) Maloney C, Sequeira E, Kelly C, Orris R, Beck J. "PubMed Central". National Center for Biotechnology Information (US). 2018; Available at: [https://www.ncbi.nlm.nih.gov/pmc/](https://www.ncbi.nlm.nih.gov/pmc/).

(8) Wishart D, Arndt D, Pon A, Sajed T, Guo AC, Djoumbou Y, et al. T3DB: The toxic exposome database. Nucleic Acids Res 2015;43(D1):D928-D934.

(9) Hastings J, Owen G, Dekker A, Ennis M, Kale N, Muthukrishnan V, et al. ChEBI in 2016: Improved services and an expanding collection of metabolites. Nucleic Acids Res 2016;44(D1):D1214-D1219.

(10) Kim S, Thiessen PA, Bolton EE, Chen J, Fu G, Gindulyte A, et al. PubChem substance and compound databases. Nucleic Acids Res 2016;44(D1):D1202-D1213.

(11) ChemAxon's Marvin Suite. 2017; Available at: <https://www.chemaxon.com/download/marvin-suite/>, 2017.

(12) Marchant CA, Briggs KA, Long A. In silico tools for sharing data and knowledge on toxicity and metabolism: Derek for windows, meteor, and vitic. Toxicol Mechan Methods 2008;18(2-3):177-187.

(13) Giacomoni F, Le Corguillé G, Monsoor M, Landi M, Pericard P, Pétéra M, et al. Workflow4Metabolomics: a collaborative research infrastructure for computational metabolomics. Bioinformatics 2015;31(9):1493.
