# Supplementary material for: BioTransformer: a comprehensive computational tool for small molecule metabolism prediction and metabolite identification
Source: J Cheminform. 2019 Jan 5;11:2. doi: 10.1186/s13321-018-0324-5 (PMC6689873; doi:10.1186/s13321-018-0324-5)
Supplement: Supplementary file 3 — Additional file 3. Phase-II-Filter-Features. [file 13321_2018_324_MOESM3_ESM.docx]

**Title**

## BioTransformer: A Comprehensive Computational Tool for Small Molecule Metabolism Prediction and Metabolite Identification

# Authors

Yannick Djoumbou-Feunang^1^, Jarlei Fiamoncini^2,3^, Alberto Gil-de-la-Fuente^4^, Russell Greiner^5,6^, Claudine Manach^2^, David S. Wishart^1,5^

# Affiliations

^1^Department of Biological Sciences, University of Alberta, Edmonton, Alberta, Canada, T6G 2E9

^2^INRA, Human Nutrition Unit, Université Clermont Auvergne, F63000 Clermont-Ferrand, France

^3^Department of Food and Experimental Nutrition, School of Pharmaceutical Sciences, University of São Paulo, São Paulo, Brazil

^4^Department of Information Technology, CEU San Pablo University, Madrid Spain

^5^Department of Computing Science, University of Alberta, Edmonton, Alberta, Canada, T6G 2E8

^6^Alberta Machine Intelligence Institute, University of Alberta, Edmonton, Alberta, Canada T6G 2E8

## The Phase II Metabolism Prediction System

Table S1 Structural features used in the Phase II filter model. All but the last seven features were selected for the PhaseII filter model.

| **Feature** | **Description** |
| --- | --- |
| nHBA | Number of Hydrogen bond acceptors |
| tpsa | Topological Polar Surface Area |
| alogp | ALogP |
| nAcidicGroups | Number of acidic groups |
| nHBD | Number of Hydrogen bond donors |
| monoisoMass | Monoisotopic mass |
| nRotBonds | Number of rotatable bonds |
| nBasicGroups | Number of basic groups |
| hBond | Number of Hydrogen bonds |
| hydroxyl_acidic | Number of acidic hydroxyl groups |
| unsubstituted_aromatic_carbon | Number of unsubstituted aromatic carbon atoms |
| carboxyl | Number of carboxyl groups |
| carbon_hydrogen_bond | Number of hydrogen bonds |
| alcohol | Number of alcohol groups |
| carboxylic_acid_or_base | Number of acidic and basic carboxyl groups |
| hydroxyl | Number of hydroxyl groups |
| unfunctionalized_methylen_group | Number of unfunctionalized methylene groups |
| 12_diol | Number of 1,2-diols |
| ewg | Number of electron withdrawing groups |
| heterocyclic_amine | Number of heterocyclic amine groups |
| imine | Number of imine groups |
| amine | Number of amine groups |
| carboxylic_ester | Number of carboxylic ester groups |
| **Feature** | **Description** |
| peroxide | Number of peroxide groups |
| heteroatom | Number of heteroatoms |
| disulfide | Number of disulphide bonds |
| aryl_halide | Number of aryl-halide groups |
| acyl_halide | Number of acyl-halide groups |
| n_metyl | Number of N-methyl groups |
| o_metyl | Number of O-methyl groups |
| aryl_nitro | Number of aryl-nitro groups |
| epoxide | Number of epoxide groups |

Table S2 SMARTS expressions of pre-filters: The first row shows the SMARTS pattern for the “glycerolipid” chemical class, used to discriminate glycerolipid compounds from potential Phase II candidates. The last 64 rows display SMARTS patterns of features that can be pre-selected as Phase II substrates and passed to the Phase II filter for prediction.

| **Feature** | **SMARTS** |
| --- | --- |
| GLYCEROLIPID | [$([OX1-,OX2H1,$([OX2](-[CX4])[CX4;R0;H2]),$([OX2]-[CX3]=[CX3]),$([OX2]-[CX3](=O)-[#1,#6])][#6;A;H2X4R0][#6;A;H1X4R0]([OX1-,OX2H1,$([OX2](-[CX4])[CX4;R0;H2]),$([OX2]-[CX3]=[CX3]),$([OX2]-[CX3](=O)-[#1,#6])])[#6;A;H2X4R0][#8]-[CX4,$([CX3]=[CX3]),$([CX3](=O)-[#1,#6])]),$([OX1-,OX2H1,$([OX2](-[CX4])[CX4;R0;H2]),$([OX2]-[CX3]=[CX3]),$([OX2]-[CX3](=O)-[#1,#6])][#6;A;H2X4R0][#6;A;H1X4R0]([#6;A;H2X4R0][OX1-,OX2H1,$([OX2](-[CX4])[CX4;R0;H2]),$([OX2]-[CX3]=[CX3]),$([OX2]-[CX3](=O)-[#1,#6])])[#8;X2]-[CX4,$([CX3]=[CX3]),$([CX3](=O)-[#1,#6])])] |
| feature_01 | [H][#8;X2][#6;A;X4]([#6])([#6,#1])[#6,#1] |
| feature_02 | [#8;H1X2]-[#6,#7,#16;a] |
| feature_03 | [#6;A;!$(C(-[NX3H2])-[CX3](=O)[OX2H1,OX1-])][#6;A;X3]([#8;H1X2])=O |
| feature_04 | [#6;a]-[#6]([#8;A;X2H1,X1-])=O |
| feature_05 | [O;X2H1][N;X3](-[#6;X4H1,X4H2,X4H3]) |
| feature_06 | [#6]-[#16;X4](=[#8;X1])(=[#8;X1])-[#7;X3;H1,H2] |
| feature_07 | [#6;a]-[N;X3;H2] |
| **Feature** | **SMARTS** |
| feature_08 | [#6]=,:1[#6]=,:[#6;R1][#7;H1v4X3]=,:[#6;R1][#6]=,:1 |
| feature_09 | [#6]-[#16;H1X2] |
| feature_10 | [#16;H1X2]-[#6;R1]=,:1[#6;R1]=,:[#6;R1][#6;R1]=,:[#6;R1][#6;R1]=,:1 |
| feature_11 | [O;X1]=[#6;X3]-[#6;X4H2]-[#6;X3]=[O;X1] |
| feature_12 | [H][#8;X2]-[#6;R1]=,:1[#6;R1]=,:[#6;R1][#6;R1](=,:[#6;R1][#6;R1]=,:1)-[#6;R1]1=,:[#6;R1][#8;R1][#6;R2]2=,:[#6;R1][#6;R1]=,:[#6;R1][#6;R1]=,:[#6;R2]2[#6;R1]1=[O;X1] |
| feature_13 | [H][#8;X2]-[#6;R1]1=,:[#6;R1][#6;R1]=,:[#6;R2]2[#6;R2](=,:[#6;R1]1)[#8;R1][#6;R1]=,:[#6;R1](-[#6;R1]=,:1[#6;R1]=,:[#6;R1][#6;R1]=,:[#6;R1][#6;R1]=,:1)[#6;R1]2=[O;X1] |
| feature_14 | [#6]!@-[#7;A;H1X3;!$([N]*~[#7,#8,#15,#16])]([#6])[#6] |
| feature_15 | [#7;X3;$([H2]),$([H1][#6;!$(C=[O,N,S])])]-[#6](-[#6X4,#7X3])=[O;X1] |
| feature_16 | [#6]=,:1[#7;H1v3X3R1][#7;R1]=,:[#6,#7][#6,#7]=,:1 |
| feature_17 | [*,#1]-[#7;v3X3R1]1[#6]=,:[#6,#7][#6,#7]=,:[#7;v3R1]1 |
| feature_18 | *-[#7]1[#6]=,:[#6;R1][#7;v3R1]=,:[#6;R1]1 |
| feature_19 | [#6]=,:1[#7;R1][#7;R1]=,:[#6][#7;v3R1]=,:1 |
| feature_20 | [#8;A;H1X2][#6]~1~[#6,#8,#7,#16]~[#6,#8,#7,#16]~[#6,#8,#7,#16]~2~[#6,#8,#7,#16;A;R2](~[#6,#8,#7,#16]~[#6,#8,#7,#16;A;R1]~[#6,#8,#7,#16;A;R2]-,=3-,=[#6,#8,#7,#16;A;R2]-,=4-,=[#6,#8,#7,#16]-,=[#6,#8,#7,#16][#6;A;H1X4][#6,#8,#7,#16]-,=4-,=[#6,#8,#7,#16][#6;A;X4;H2,H1][#6,#8,#7,#16]~2-,=3)~[#6,#8,#7,#16]~1 |
| feature_21 | [#8;A;H1X2][#6]~1~[#6,#8,#7,#16;A;R1]~[#6,#8,#7,#16;A;R2]~2-,=[#6,#8,#7,#16;A;R2]-,=3-,=[#6,#8,#7,#16]-,=[#6,#8,#7,#16][#6;A;H1X4][#6,#8,#7,#16]-,=3-,=[#6,#8,#7,#16][#6;A;X4;H2,H1][#6,#8,#7,#16]~2~[#6,#8,#7,#16]~2~[#6,#8,#7,#16]~[#6,#8,#7,#16]~[#6,#8,#7,#16]~[#6,#8,#7,#16]~[#6,#8,#7,#16;A;R2]~1~2 |
| feature_22 | [#8;A;H1X2][#6;A;X4]1([#1,CX4H3R0])[#6,#8,#7,#16]-,=[#6,#8,#7,#16]-,=[#6,#8,#7,#16;A;R2]-,=2-,=[#6,#8,#7,#16;A;R2]~3~[#6,#8,#7,#16;A;R1]~[#6,#8,#7,#16]~[#6,#8,#7,#16;A;R2]~4~[#6,#8,#7,#16]~[#6,#8,#7,#16]~[#6,#8,#7,#16]~[#6,#8,#7,#16]~[#6,#8,#7,#16]~4~[#6,#8,#7,#16]~3[#6;A;X4;H2,H1][#6,#8,#7,#16]-,=[#6,#8,#7,#16]1-,=2 |
| feature_23 | [$([#6][N;X4+]([#6])([#6])[#8;X1-]),$([#6][#7;X3+](=,:[#6])-[#8;X1-])] |
| feature_24 | [H][#8]-[#6;R1]=,:1[#6;R1]=,:[#6;R1][#6]-2=,:[#6]([#6;R1]=,:1)-[#6;R1]-[#6;R1]-[#6]-1-[#6]-3-[#6;R1]-[#6;R1]-[#6;R1]-[#6]-3-[#6;R1]-[#6;R1]-[#6]-2-1 |
| feature_25 | [#6;A;H2X4;!$(C([OX2H])[O,S,#7,#15])][#8;H1X2] |
| feature_26 | [H][#8][#6;A;H1X4;!$(C([OX2H])[O,S,#7,#15])]([H])([#6])[#6] |
| feature_27 | [H][#8]-[#6;R1]=,:1[#6;R1]=,:[#6;R1]([H])[#6;R1]([H])=,:[#6;R1](!@-[#6])[#6;R1]=,:1 |
| feature_28 | [H][#6;R1]1=,:[#6;R1][#6;R1](!@-[#8;H1X2R0])=,:[#6;R1][#6;R1]([H])=,:[#6;R1]1!@-[#6] |
| feature_29 | [H][#8]-[#6]~1~[#6,#8,#7,#16]~[#6,#8,#7,#16]~[#6,#8,#7,#16]~2~[#6,#8,#7,#16;A;R2](~[#6,#8,#7,#16]~[#6,#8,#7,#16;A;R1]~[#6,#8,#7,#16;A;R2]~3-,=[#6,#8,#7,#16;A;R2]-,=4-,=[#6,#8,#7,#16]-,=[#6,#8,#7,#16]-,=[#6,#8,#7,#16;A]-,=[#6,#8,#7,#16]-,=4-,=[#6,#8,#7,#16]~[#6,#8,#7,#16;A]~[#6,#8,#7,#16]~2~3)~[#6,#8,#7,#16]~1 |
| **Feature** | **SMARTS** |
| feature_30 | [H][#8]C1([H])[#6,#8,#7,#16]~[#6,#8,#7,#16;A;R2]~2~[#6,#8,#7,#16]~[#6,#8,#7,#16]~[#6,#8,#7,#16]~[#6,#8,#7,#16]~[#6,#8,#7,#16]~2~[#6,#8,#7,#16]~2~[#6,#8,#7,#16;A]~[#6,#8,#7,#16]-,=[#6,#8,#7,#16]-,=3-,=[#6,#8,#7,#16;A]-,=[#6,#8,#7,#16]-,=[#6,#8,#7,#16]-,=[#6,#8,#7,#16;A;R2]-,=3-,=[#6]1~2 |
| feature_31 | [H][#8]C1([H])[#6]~[#6,#8,#7,#16]~2~[#6,#8,#7,#16]~3~[#6,#8,#7,#16]~[#6,#8,#7,#16]~[#6,#8,#7,#16]~[#6,#8,#7,#16]~[#6,#8,#7,#16;A;R2]~3~[#6,#8,#7,#16]~[#6,#8,#7,#16;A;R1]~[#6,#8,#7,#16;A;R2]~2-,=[#6,#8,#7,#16;A;R2]-,=2-,=[#6,#8,#7,#16]-,=[#6,#8,#7,#16]-,=[#6,#8,#7,#16;A]-,=[#6]1-,=2 |
| feature_32 | [H][#7;X3+0,X4+;!$([N]~[!#6]!$([N]*~[#7,#8,#15,#16])]-[#6;a] |
| feature_33 | [H][#7;X3+0,X4+;!$([N]~[!#6])][#7;A;X3]([H])[#6;a] |
| feature_34 | [#6]-[#16;X2][#6;A;H2X4][#6;A@H;X4]([#7;A;H2X3])[#6](-[#8-])=O |
| feature_35 | [#6;a][#6;A;H2X4][#6;A;H2X4][#7;A;X3+0,X4+;!$([N]~[!#6]!$([N]*!@[#7,#8,#15,#16])] |
| feature_36 | [#8;A;X2H1,X1-][#7;A;H1X3]!@-[#6]=,:1[#6]=,:[#6][#6]=,:[#6][#6]=,:1 |
| feature_37 | [H][#8]-[#7;X3](-[#6;X3]([#6;A;H3X4])=[O;X1])!@-[#6;X3]-1=[#6]-[#6]=[#6]-[#6]=[#6]-1 |
| feature_38 | [#6]-[#6;X4]-1-[#8]-[#6;X4]-1-[#1,#6,#7,#8] |
| feature_39 | [#6][S;X2H1] |
| feature_40 | [#8;X2]-1-[#6;X3]-2=[#6;X3]-1-[#6]=[#6]-[#6]=[#6]-2 |
| feature_41 | [H][C;X4]([#1,#6])([F,Cl,Br,I])[*,#1] |
| feature_42 | [H][#6;X3](-[F,Cl,Br,I])=[#6;X3]([*,#1])-[*,#1] |
| feature_43 | [#6]-[#6;X3](Cl)=[O;X1] |
| feature_44 | [#6]-[#7]=[#6]-[H1X2;O,S] |
| feature_45 | [H][#6;X3R1]-1=[#6;X3R1]-[#6;R1](=[!#1!#6;R0;OX1,NX2])-[#6;X3]=[#6;X3]-[#6;R1]-1=[O;X1] |
| feature_46 | [H][#6;X3](-*)=[#6;X3]([$([CX3;$([R0][#6]),$([H1R0])](=[OX1])[OX2][#6;!$(C=[O,N,S])]),$([$([CX3H][#6]),$([CX3H2])]=[OX1]),$([#6][CX3](=[OX1])[#6])])-[*,#1] |
| feature_47 | [#6]-[#7;X2]=[C;X2]=[O;X1] |
| feature_48 | [#6]-[#7;X2]=[C;X2]=[S;X1] |
| feature_49 | [!#1!#6;F,Cl,Br,I,$([N+]),$([#7;X3+](-[#8;X1-])=[O;X1]),$([#6]~[C;X4](F)(F)F),$([C;X4](Cl)(Cl)Cl),$([S;X4]([#8;A;X1-,X2H1])(=O)=O),$([#6]=O),$(C#N)]~[#6]1=,:[#6][#6]=,:[#6](-[F,Cl,Br,I])[#6](~[#1,!#6;F,Cl,Br,I,$([N+]),$([#7;X3+](-[#8;X1-])=[O;X1]),$([#6]~[C;X4](F)(F)F),$([C;X4](Cl)(Cl)Cl),$([S;X4]([#8;A;X1-,X2H1])(=O)=O),$([#6]=O),$(C#N)])=,:[#6]1 |
| feature_50 | [#1,!#6;F,Cl,Br,I,$([N+]),$([#7;X3+](-[#8;X1-])=[O;X1]),$([#6]~[C;X4](F)(F)F),$([C;X4](Cl)(Cl)Cl),$([S;X4]([#8;A;X1-,X2H1])(=O)=O),$([#6]=O),$(C#N)]-[#6]=,:1[#6]=,:[#6][#6](-[F,Cl,Br,I])=,:[#6]([!#1!#6;F,Cl,Br,I,$([N+]),$([#7;X3+](-[#8;X1-])=[O;X1]),$([#6]~[C;X4](F)(F)F),$([C;X4](Cl)(Cl)Cl),$([S;X4]([#8;A;X1-,X2H1])(=O)=O),$([#6]=O),$(C#N)])[#6]=,:1 |
| **Feature** | **SMARTS** |
| feature_51 | [$([H][#8]-[#6]=,:1[#6]=,:[#6](-[F,Cl,Br,I,$([N+]),$([#7;X3+](-[#8;X1-])=[O;X1]),$([#6]~[C;X4](F)(F)F),$([C;X4](Cl)(Cl)Cl),$([S;X4]([#8;A;X1-,X2H1])(=O)=O),$([#6]=O),$(C#N)])[#6]=,:[#6][#6]=,:1-[F,Cl,Br,I]),$([H][#8]-[#6]=,:1[#6]=,:[#6][#6](-[F,Cl,Br,I,$([N+]),$([#7;X3+](-[#8;X1-])=[O;X1]),$([#6]~[C;X4](F)(F)F),$([C;X4](Cl)(Cl)Cl),$([S;X4]([#8;A;X1-,X2H1])(=O)=O),$([#6]=O),$(C#N)])=,:[#6][#6]=,:1-[F,Cl,Br,I]),$([H][#8]-[#6]1=,:[#6][#6](-[F,Cl,Br,I,$([N+]),$([#7;X3+](-[#8;X1-])=[O;X1]),$([#6]~[C;X4](F)(F)F),$([C;X4](Cl)(Cl)Cl),$([S;X4]([#8;A;X1-,X2H1])(=O)=O),$([#6]=O),$(C#N)])=,:[#6][#6](-[F,Cl,Br,I])=,:[#6]1),$([H][#8]-[#6]1=,:[#6](-[F,Cl,Br,I,$([N+]),$([#7;X3+](-[#8;X1-])=[O;X1]),$([#6]~[C;X4](F)(F)F),$([C;X4](Cl)(Cl)Cl),$([S;X4]([#8;A;X1-,X2H1])(=O)=O),$([#6]=O),$(C#N)])[#6]=,:[#6][#6]=,:[#6]1-[F,Cl,Br,I]),$([H][#8]-[#6]=,:1[#6]=,:[#6][#6](-[F,Cl,Br,I])=,:[#6](-[F,Cl,Br,I,$([N+]),$([#7;X3+](-[#8;X1-])=[O;X1]),$([#6]~[C;X4](F)(F)F),$([C;X4](Cl)(Cl)Cl),$([S;X4]([#8;A;X1-,X2H1])(=O)=O),$([#6]=O),$(C#N)])[#6]=,:1),$([H][#8]-[#6]1=,:[#6][#6]=,:[#6][#6](-[F,Cl,Br,I,$([N+]),$([#7;X3+](-[#8;X1-])=[O;X1]),$([#6]~[C;X4](F)(F)F),$([C;X4](Cl)(Cl)Cl),$([S;X4]([#8;A;X1-,X2H1])(=O)=O),$([#6]=O),$(C#N)])=,:[#6]1-[F,Cl,Br,I])] |
| feature_52 | [H][#6;R1]-1=[#6]-[#6]=[#6;R1]-[#6;R1](=[O;X1R0])-[#6;R1]-1=[O;X1R0] |
| feature_53 | [H][#6]-1=[#6]-[#6;R1](=[O;X1R0])-[#6;R1]=[#6;R1]-[#6;R1]-1=[O;X1R0] |
| feature_54 | [F,Cl,Br,I]-[#6;R1]=,:1[#7;R1]=,:[#6;R1][#7;R1]=,:[#6;R1][#7;R1]=,:1 |
| feature_55 | [H][#6;R1]1=,:[#6;R1][#6;R1](-[R0;F,Cl,Br,I,$([N+]),$([#7;X3+](-[#8;X1-])=[O;X1]),$([#6]~[C;X4](F)(F)F),$([C;X4](Cl)(Cl)Cl),$([S;X4]([#8;A;X1-,X2H1])(=O)=O),$([#6]=O),$(C#N)])=,:[#6;R1](-[R0;F,Cl,Br,I,$([N+]),$([#7;X3+](-[#8;X1-])=[O;X1]),$([#6]~[C;X4](F)(F)F),$([C;X4](Cl)(Cl)Cl),$([S;X4]([#8;A;X1-,X2H1])(=O)=O),$([#6]=O),$(C#N)])[#6;R1]=,:[#6;R1]1-[R0;F,Cl,Br,I,$([N+]),$([#7;X3+](-[#8;X1-])=[O;X1]),$([#6]~[C;X4](F)(F)F),$([C;X4](Cl)(Cl)Cl),$([S;X4]([#8;A;X1-,X2H1])(=O)=O),$([#6]=O),$(C#N)] |
| feature_56 | [H][#6;R1]=,:1[#6;R1](-[R0;F,Cl,Br,I,$([N+]),$([#7;X3+](-[#8;X1-])=[O;X1]),$([#6]~[C;X4](F)(F)F),$([C;X4](Cl)(Cl)Cl),$([S;X4]([#8;A;X1-,X2H1])(=O)=O),$([#6]=O),$(C#N)])=,:[#6;R1][#6;R1](-[R0;F,Cl,Br,I,$([N+]),$([#7;X3+](-[#8;X1-])=[O;X1]),$([#6]~[C;X4](F)(F)F),$([C;X4](Cl)(Cl)Cl),$([S;X4]([#8;A;X1-,X2H1])(=O)=O),$([#6]=O),$(C#N)])=,:[#6;R1][#6;R1]=,:1-[R0;F,Cl,Br,I,$([N+]),$([#7;X3+](-[#8;X1-])=[O;X1]),$([#6]~[C;X4](F)(F)F),$([C;X4](Cl)(Cl)Cl),$([S;X4]([#8;A;X1-,X2H1])(=O)=O),$([#6]=O),$(C#N)] |
| feature_57 | [H][#8;X2]-[#6;X3](-[#6;$([#7;A;R0]-,=[#6;A;R0]-,=[#6;A;R0]-,=[#6;A;R0]),$([#7]-[#6;A;X3]=[#6;A]-[#6]=,:1[#6]=,:[#6][#6]=,:[#6][#6]=,:1)])=[O;X1] |
| feature_58 | [c;a;R1]-[#6;X3](-[OX2H1,OX1-])=O |
| feature_59 | [H][#8;A;X2][#6;R1]1=,:[#6;R1](-[*,#1;!$([OX2H1])])[#6;R1](-[*,#1])=,:[#6;R1](-[*,#1])[#6;R1](-[*,#1;!$([OX2H1])])=,:[#6;R1]1[#8;A;X2][H] |
| feature_60 | [H][#8;A;X2][#6;R1]1=,:[#6;R1]([H])[#6;R1]([H])=,:[#6;R1](-[!#8])[#6;R1]([H])=,:[#6;R1]1[#8;A;X2][H] |
| feature_61 | [H][#8]-[#6]1=,:[#6]([H])[#6]([H])=,:[#6](-[!#8])[#6]([H])=,:[#6]1-[#8][H] |
| feature_62 | [#6]-[#16;R0;X2H1+0,X1-1] |
| feature_63 | [#16;A;H1X2][c;R1]1[n;R1][c;R1][n;R1][c;R2]2[n;R1][c;R1][n;R1][c;R2]12 |
| feature_64 | [#6]-[#16;X2+0;R0]-[#6] |
